# Supplementary material for: Impact of sharing electronic health records with patients on the quality and safety of care: a systematic review and narrative synthesis protocol
Source: BMJ Open. 2018 Aug 13;8(8):e020387. doi: 10.1136/bmjopen-2017-020387 (PMC6091908; doi:10.1136/bmjopen-2017-020387)
Supplement: Author's manuscript [file bmjopen-2017-020387.draft_revisions.pdf]

BMJ Open is committed to open peer review. As part of this commitment we make the peer review history of every article we publish publicly available.

When an article is published we post the peer reviewers' comments and the authors' responses online. We also post the versions of the paper that were used during peer review. These are the versions that the peer review comments apply to.

The versions of the paper that follow are the versions that were submitted during the peer review process. They are not the versions of record or the final published versions. They should not be cited or distributed as the published version of this manuscript.

BMJ Open is an open access journal and the full, final, typeset and author-corrected version of record of the manuscript is available on our site with no access controls, subscription charges or pay-per-view fees (http://bmjopen.bmj.com).

If you have any questions on BMJ Open's open peer review process please email info.bmjopen@bmj.com

BMJ Open

Applying an analytical framework to map the impact of electronic data sharing on quality of care and safety: protocol for a systematic review

Journal:	*BMJ Open*
Manuscript ID	bmjopen-2017-020387
Article Type:	Protocol
Date Submitted by the Author:	01-Nov-2017
Complete List of Authors:	Neves, Ana Luisa; Imperial College London, Center for Health Policy, Institute of Global Health Innovation<br>Carter, Alexander; Imperial College London, Center for Health Policy, Institute of Global Health Innovation<br>Freise, Lisa; Imperial College London, Center for Health Policy, Institute of Global Health Innovation<br>Laranjo, Liliana; Macquarie University Faculty of Science, Australian Institute of Health Innovation; Universidade Nova de Lisboa, Centro de Investigação em Saúde Pública, Escola Nacional de Saúde Pública<br>Darzi, Ara; Imperial College London, Center for Health Policy, Institute of Global Health Innovation<br>Mayer, Erik; Imperial College London, Center for Health Policy, Institute of Global Health Innovation
Keywords:	Health informatics < BIOTECHNOLOGY & BIOINFORMATICS, Information technology < BIOTECHNOLOGY & BIOINFORMATICS, Quality in health care < HEALTH SERVICES ADMINISTRATION & MANAGEMENT, Health policy < HEALTH SERVICES ADMINISTRATION & MANAGEMENT, Health & safety < HEALTH SERVICES ADMINISTRATION & MANAGEMENT

SCHOLARONE™
Manuscripts

Applying an analytical framework to map the impact of electronic data sharing on quality of care and safety: protocol for a systematic review

Authors: Ana Luisa Neves[1] (ALN) - ana.luisa.neves14@imperial.ac.uk, Alexander Carter (AC) - a.w.carter@imperial.ac.uk, Lisa Freise[1] (LF) - l.freise16@imperial.ac.uk, Liliana Laranjo[2] - liliana.laranjo@mq.edu.au, Ara Darzi[1] (AD) - a.darzi@imperial.ac.uk, Erik Mayer[1] (EM) - e.mayer@imperial.ac.uk

[1]Center for Health Policy, Institute of Global Health Innovation, Imperial College London, London, UK
[2]Australian Institute of Health Innovation, Centre for Health Informatics, Macquarie University, Sidney, Australia

For peer review only

Corresponding author

Name: Ana Luisa Neves

Address: Centre for Health Policy, Room 1035/7, QEQM Wing, St Mary's Campus, London W2 1NY

E-mail: ana.luisa.neves14@ic.ac.uk

Telephone: 02033127259

Fax number: 02033126309

Keywords [MeSH terms]: Electronic Health Records, Patient Access to Records, Patient-Centered Care, Quality of Health Care, Patient Safety

Word count: 2145 words

Abstract

Introduction: Providing patients with access to Electronic health records (EHR) has emerged as a promising solution to improve quality of care and safety. However, there is a considerable gap between the predicted and demonstrated benefits of these interventions. As the efforts to develop and implement EHR-based data sharing platforms mature and scale up worldwide, there is a need to evaluate the impact of these interventions and to weigh their relative risks and benefits, in order to inform evidence-based health policies. The aim of this work is to systematically characterise and appraise the demonstrated benefits of EHR-based data sharing interventions, by mapping them across the six domains of quality of care of the Institute of Medicine analytical framework (i.e. patient-centredness, effectiveness, efficiency, timeliness, equity and safety).

Methods and analysis: CINAHL, Cochrane, Embase, HMIC, Medline/PubMed and PsycINFO databases will be searched from January 1997 to August 2017. Primary outcomes will include measures related with the six domains of quality of care of the IOM analytical framework (i.e. patient-centredness, effectiveness, efficiency, timeliness, equity and safety). Valid studies will be assessed for their quality using the Cochrane Risk of Bias Tool by two independent researchers and disagreements will be resolved by a third person. A narrative synthesis will be conducted for all the included studies. The body of evidence will be summarised in a Summary of Findings table and the strength of the body of evidence will be assessed according to the GRADE criteria. Results will be used to develop a comprehensive framework of the demonstrated benefits of EHR-based data sharing interventions, and subgroup analysis will be performed by domain of quality of care domain and by time scale (*i.e.* short-, medium- or long-term impact). This protocol was registered in PROSPERO (CRD42017070092).

Ethics and dissemination: Not applicable.

For peer review only

Strengths and limitations of this study

- Comprehensive characterisation of EHR-based interventions used to share medical data with patients
- Summary and appraisal of existing evidence on the potential benefits EHR-based data sharing interventions, grouped by domain of quality of care and by time scale (*i.e.* short-, medium- or long-term impact)
- Development of a framework to customise informed decisions in health policies
- Expected limitations include the heterogenous nature of the outcomes assessed and the potentially reduced sample size in subgroup analyses, which may negatively impact the statistical power in data synthesis.

For peer review only

Introduction

Although patients have had the legal right to access their health records since 1998, access to paper-based health records is mediated by health professionals and data controllers, through a cumbersome procedural process (1). Additionally, as health information is fragmented between different organisations and levels of care, data access requests are often unable to provide a comprehensive health history record (2, 3).

In the last decade, electronic health records (EHRs), have emerged as a promising solution to enhance patients' access to centralised medical information (4). The adoption of EHRs by primary care practices, hospitals and other healthcare organizations has steadily increased in the last years. In England, the percentage of General Practice surgeries that allowed patients to access their medical records online increased from 3% to 97% between April 2014 and February 2016 (5). Various EHR-based platforms are currently used to share health information with patients, including direct online access, with or without patient-provider communication systems (6, 7) and health maintenance reminders (8, 9). As these efforts mature and scale up worldwide, there is a need to evaluate the impact of EHR-based data sharing interventions, in order to weigh their relative risks and benefits and inform evidence-based health policies.

The Institute of Medicine (IOM) identified six domains of health care quality: patient-centredness, effectiveness, efficiency, timeliness, equity and safety (10). Patient-centredness ensures that the care provided respects and responds to individual patient preferences, needs and values, thus incorporating them in clinical decisions (10, 11) Health care shall provide evidence-based services, which can be ultimately expressed as improvements in health outcomes (*i.e.* effectiveness) (12), while ensuring that potential harms are avoided (*i.e.* patient safety). Other aspects of quality of delivery of care include the minimisation of the waste of human, physical or economical resources (*i.e.* efficiency), the reduction of waits and harmful delays (*i.e.* timeliness) and the reduction of avoidable differences on the delivery of care between different groups of health care users (*i.e. equity)* (10, 13, 14).

Providing patients with access to their health records has been linked to theorised benefits in four major domains of health care quality: patient-centredness, effectiveness, safety and efficiency (15-17). However, despite the growing body of evidence on the theorised benefits of EHR-data sharing with patients on these domains, there is still a considerable gap between the predicted and demonstrated benefits of these interventions (18).

In order to analyse the effect of providing patients access to their medical records on quality outcomes, Davis-Giardina T. et al performed a systematic review including studies published between 1970 and 2012 (19). According to this work, a limited amount of evidence suggests that access to medical records

For peer review only - http://bmjopen.bmj.com/site/about/guidelines.xhtml

improves patient satisfaction and enhances patient-provider communication (19). Similarly, a systematic review from de Lusignan *et al.* reported that providing patients online access to their EHR increased convenience and satisfaction (20).

Conversely, no clear benefits were found on effectiveness (19). Until 2012, only a few studies evaluated the impact on effectiveness studies, most focussing on type 2 diabetes and with inconsistent results. Tenforde *et al* showed that providing access to medical records was associated with lower glycated haemoglobin A1C values (21); however, no significant effect was found in three other studies assessing diabetes-specific effectiveness measure (22, 23). One of the limitations of this review consists in the inclusion of studies evaluating both EHR and non-EHR based interventions - and this heterogeneity might mask potential EHR-specific potential benefits. Furthermore, as pointed by the authors, the paucity of papers published up to that date resulted in a tendency to include small and methodologically less robust studies, thus increasing the risk of selective reporting and/or publication bias (19). Mold F. *et al* also performed a systematic review assessing the impact of providing patients with access to their EHRs; based in studies published between 1999 and 2012, this work found a positive influence in patient safety (24).

However, the authors were unable to find a consistent beneficial effect on efficiency measures (*i.e.* number of face-to-face visits and telephone appointments) in both reviews (24) (19). While some studies reported an increase in the number of face-to-face consultations (6,25), others document a decrease (9,26-27). Similarly, inconsistent results were found regarding the impact on telephone consultations: only one study reported a decline in the total number (28), whilst six other studies reported either no change or an increase (7, 25-27, 29, 30). It is important to note, however, that most of the included studies assessing efficiency measures included in this review showed a high risk of bias, mostly related to either unclear or absent blinding methods (24).

The landmark reviews of Mold F. *et al* (24) and Davis-Giardina T. *et al* (19) provide a comprehensive characterisation of the literature published until 2012, highlighting the paucity and the scientific limitations of the evidence published until that date. Although both were unable to demonstrate clear benefits on efficiency and effectiveness measures, resources continue being allocated to EHR-based data sharing interventions and platforms, renewing the interest on this evidence gap. As consequence of these efforts, it is plausible that studies performed in the last 5 years can provide further clarification for this evidence gap. Furthermore, these reviews do not address other domains of quality of care, as timeliness or equity.

For peer review only

This review will expand on the above-mentioned work, in order to identify recent methodological and scientific progress until June 2017. Following the PRISMA-P checklist as guidance (31), we propose a systematic and reproducible strategy to query the literature on the demonstrated benefits of EHR-based data sharing and present the results in a comprehensive framework of health care quality measures.

Research aims

The main objectives of this review are: 1) to systematically characterise EHR-based data sharing interventions published between 2000-2017; 2) to assess the demonstrated benefits of these interventions on patient-centredness, effectiveness, safety, efficiency, timeliness and equity, compared to usual care (no intervention). As secondary aim, we will develop a conceptual model integrating the contribution of these interventions in short-, medium- and long-term perspectives (Figure 1).

Methods and analysis

Search strategy

The search strategy will be performed using resources that enhance methodological transparency and improve the reproducibility of the results and evidence synthesis. A search of the literature from the last 20 years (January 1997 – August 2017) will be performed on CINAHL, Cochrane, Embase, HMIC, Medline/PubMed and PsycINFO. Search strings (Table 1) will combine free terms and controlled vocabulary, whenever supported. Language restrictions will be applied and articles in English will be included. The reference lists of relevant articles will also be screened to ensure all eligible studies are captured. Authors of protocols potentially meeting inclusion criteria will be contacted to provide further information about the progress of the corresponding trial.

Study selection criteria

A summary of the participants, interventions, comparators and outcomes (PICO) considered, as well as the type of studies included, is provided in Table 2.

The systematic review will focus on studies on adult subjects, including both patients and carers (mean age of study sample ≥ 16 years). The systematic review does not focus on a particular disease area or health system setting as it intends to comprehensively characterise the scope of EHR-based interventions.

For peer review only

Studies describing EHR-based data sharing interventions, either isolated or as part of a multicomponent interventions, will be included. "*EHR-based data sharing intervention*" will be defined as a data sharing intervention involving: 1) web-based patient access to EHR; 2) EHR-based health reminders / messaging or 3) online patient-provider communication systems (health information exchange platforms). Studies focusing on health reminders only (not EHR-based) or appointment reminders will not be considered. The comparator will be 'no intervention' (*e.g.* usual care).

Primary outcomes will include any measure related to a) patient-centredness (*e.g.* patient-reported experience measures (PREMs), b) effectiveness (*e.g.* health outcomes); c) patient safety (*e.g.* identification of medication discrepancies); d) efficiency (*e.g.* economic evaluation measures and proxies; including service costs, number of consultations/admissions, e) timeliness (*e.g.* waiting lists, time-to-treatment) and f) equity (*e.g.* discrepancies in quality measures between different groups of patients). Studies that only report cognitive outcomes (*e.g.* intent), motivational outcomes or other subjective psychological measures will be excluded. The types of study considered in this systematic review will be a) randomised controlled trials; b) cluster randomised trials; c) quasi-experimental studies; d) case-control studies and e) cohort studies. Systematic reviews will also be included to ensure that relevant articles will be captured from their respective reference lists.

Screening and data extraction

Quantitative studies will be independently assessed by three reviewers and reported using the Preferred Reporting Items for Systematic Reviews and Meta-Analyses (PRISMA-P) flow diagram (31). Initial screening of studies will be based on the information contained in their titles and abstracts and will be conducted by two independent investigators. Full-paper screening will be conducted by the same independent investigators. Cohen's kappa will be used to measure inter-coder agreement in each screening phase. When there are doubts regarding inclusion or exclusion, a third investigator will be involved in the decision. Two independent investigators will extract information from the included studies into a standardized form. The data collected for each study will include: name of the first author, year of publication, technology, intervention components and characteristics, study duration, participants' and setting characteristics, outcomes and retention rates. Two investigators will review the abstraction form for consistency. Disagreements will be resolved by a third investigator.

For peer review only

Quality assessment

Valid studies will then be assessed for their quality using the Cochrane Risk of Bias Tool (32), that assesses the following study-level aspects: a) randomisation sequence allocation; b) allocation concealment; c) blinding; d) completeness of outcome data and e) selective outcome reporting. Two independent reviewers will score the selected studies and disagreements will be resolved by a third person.

Descriptive analysis and meta-analysis

A narrative synthesis will be conducted for all the included studies. Parallel-group trials that are deemed comparable in relevant ways will be pooled together for a summary effect. Whenever possible, continuous and dichotomous outcomes will be pooled together for meta-analysis purposes. All effect sizes will be transformed into a common metric, in order to make them comparable across studies — the bias-corrected standardized difference in means (Hedges' g) — classified as positive when in favor of the intervention and negative when in favor of the control. Heterogeneity will be assessed using I^2. The presence of publication bias will be evaluated by use of a funnel plot and the Duval and Tweedie's trim and fill method (33).

We will also explore the cause of any observed statistical heterogeneity using subgroup analysis. Planned subgroup analysis will be performed by domain of quality of care, time scale (*i.e.* short-, medium- or long-term impact) and risk of bias (low *versus* high, as assessed by the Cochrane Risk of Bias Tool) (32). Depending on the amount of information retrieved, subgroup analysis will also be performed for specific diseases. The body of evidence will be summarised in a Summary of Findings table and the strength of the body of evidence will be assessed according to GRADE criteria (34).

Amendments

Any amendments to this protocol will be documented with reference to saved searches and analysis methods, which will be recorded in bibliographic databases (Ovid), Endnote, and Excel templates for data collection and synthesis.

For peer review only

Discussion

As the implementation of EHR-based data sharing interventions scales up worldwide, the systematic evaluation of the impact of such interventions emerges as a priority research topic.

One of the strengths of the proposed study is to apply a reproducible and transparent procedure for systematic review of the literature. In this protocol, we clearly describe the types of studies, participants, interventions, and outcomes that will be included, as well as the data sources, search strategy, data extraction methods (including quality assessment) and methods of combining data (35). By publishing the research protocol, we reinforce the clarity of the strategy and minimise the risk of bias, namely selective outcome reporting (32). Second, we will focus solely on the impact of EHR-based studies, increasing the sensitivity to detect specific benefits of this type of intervention. Third, for the first time, we aim to comprehensively evaluate the benefits of these interventions in a wide range of domains of quality of care, as defined by the IOM. This framework shall provide high-level information to inform, support and customise decisions in health policies.

Potential limitations of this study include the heterogeneity of measures and outcomes evaluated and the potentially reduced number of studies in subgroup analyses, which may negatively influence the statistical power in data synthesis.

For peer review only

Acknowledgements

We thank Jacqueline Cousins (information specialist at Imperial College London) for the support improving the composition of the search terms and procedural aspects of the search strategy.

Ethics and dissemination

Not applicable.

Authors' contributions

ALN, AC and EKM conceptualised this research. ALN, AC and LL designed the protocol. ALN and LF defined the concepts and search items. EKM and AD contributed to the conceptualisation and commented on the multiple versions of the protocol. The manuscript was written by ALN with contributions from all authors.

Funding statement

This work is supported by the Sowerby Program and by the National Institute for Health Research (NIHR) Patient Safety Translation Research Centre. The views of the authors do not necessarily reflect those of the NHS, NIHR or the Department of Health.

Competing interests' statement

We declare no conflict(s) of interest associated with this research.

Data sharing statement

No additional data relevant to this protocol are available for distribution. All relevant data and search terms are published in this manuscript.

For peer review only

Table 1. Concepts and search items

**Database**	**Search items**
**CINAHL *via* EBSCO**	1. (((electronic* or online or on-line or digital*) N1 (health record* or medical record* or personal record* or patient record*)) or EHR# or EMR# or ephr#)<br>2. ((information or data) N4 (shar* or exchang*)) or HIE or HIEs or access*)<br>3. #1 and #2<br>4. (((experience or satisfaction) N4 (patient* or consumer* or client* or survey or questionnaire*)) or PREM* or patient-reported experience measure*)<br>5.(effectiveness or health outcome*)<br>6. (patient N1 (safety or harm)) or safety manag* or accident prevent* or error* or medication reconcil* or near miss*<br>7. (efficiency or economic* or cost* or expenditure* or charge* or fee*1 or (number N1 appointment*) or (number adj2 admission*) or (number N1 telephone visit*))<br>8. waiting lists or timeliness or time-to-treatment<br>9. health equity<br>10. #4 or #5 or #6 or #7 or #8 or #9<br>13. 3 and 10
**Cochrane *via* url:**<br>http://onlinelibrary.wiley.com/cochranelibrary/search/advanced	1. (((electronic* or online or on-line or digital*) near/1 (health record* or medical record* or personal record* or patient record*)) or EHR or EHRs or EMR or EMRs or ephr or ephrs)<br>2. Electronic Health Records [MesH]<br>3. #1 or #2<br>4. (((information or data) near/4 (shar* or exchang*)) or HIE or HIES or access*)<br>5. Information Dissemination [MesH]<br>6. #4 or #5<br>7. #3 and #6<br>8. (((experience or satisfaction) near/4 (patient* or consumer* or client* or survey or questionnaire*)) or PREM* or patient-reported experience measure*)<br>9. (effectiveness or health outcome*)<br>10. (patient near/1 safety) or (patient near/1 harm) or safety manag* or accident prevent* or error* or medication reconcil*<br>11. (efficiency or economic* or cost* or expenditure* or charge* or fee* or (number near/1 appointment*) or (number near/1 admission*) or (number near/1 telephone visit*))<br>12. time-to-treatment or timeliness

For peer review only

	13. waiting lists [MesH]<br>14. health equity [MesH]<br>15. #8 or #9 or #10 or #11 or #12 or #13 or #14<br>17. 7 and 15
**Embase *via* Ovid**	1. (((electronic* or online or on-line or digital*) adj2 (health record* or medical record* or personal record* or patient record*)) or EHR? or EMR? or ephr?)<br>2. Electronic health record/<br>3. 1 or 2<br>4. (((information or data) adj5 (shar* or exchang*)) or HIE*2 or access*)<br>5. Information dissemination/<br>6. 4 or 5<br>7. 3 and 6<br>8. (((experience or satisfaction) adj5 (patient* or consumer* or client* or survey or questionnaire*)) or PREM* or patient-reported experience measure*)<br>9. (effectiveness or health outcome*) 10. (patient adj2 (safety or harm)) or safety manag* or accident prevent* or error* or medication reconcil* or near miss*<br>11. (efficiency or economic* or cost* or expenditure* or charge* or fee*1 or (number adj2 appointment*) or (number adj2 admission*) or (number adj2 telephone visit*))<br>12. waiting list* or time to treatment/ or timeliness<br>13. health equity/<br>14. 8 or 9 or 10 11 or 12 or 13<br>15. 7 and 14
**HMIC *via* Ovid**	1. (((electronic* or online or on-line or digital*) adj2 (health record* or medical record* or personal record* or patient record*)) or EHR? or EMR? or ephr?)<br>2. Electronic patient records/<br>3. 1 or 2<br>4. (((information or data) adj5 (shar* or exchang*)) or HIE*2 or access*)<br>5. Information exchange/<br>6. 4 or 5<br>7. 3 and 6<br>8. (((experience or satisfaction) adj5 (patient* or consumer* or client* or survey or questionnaire*)) or PREM* or patient-reported experience measure*)<br>9. (effectiveness or health outcome*) 10. (patient adj2 (safety or harm)) or safety manag* or accident prevent* or error* or medication reconcil* or near miss*<br>11. (efficiency or economic* or cost* or expenditure* or charge* or fee*1 or (number adj2 appointment*) or (number adj2 admission*) or

For peer review only

	(number adj2 telephone visit*))<br>12. waiting lists/ or patient waiting time or timeliness<br>13. health inequalities/ or equity<br>14. 8 or 9 or 10 or 11 or 12 or 13<br>15. 7 and 14
**Medline via Ovid**	1. (((electronic* or online or on-line or digital*) adj2 (health record* or medical record* or personal record* or patient record*)) or EHR? or EMR? or ephr?)<br>2. Electronic Health Records/<br>3. 1 or 2<br>4. (((information or data) adj5 (shar* or exchang*)) or HIE*2 or access*<br>5. Information Dissemination/<br>6. 4 or 5<br>7. 3 and 6<br>8. (((experience or satisfaction) adj5 (patient* or consumer* or client* or survey or questionnaire*)) or PREM* or patient-reported experience measure*)<br>9. (effectiveness or health outcome*)<br>10. ((patient adj2 (safety or harm)) or safety manag* or accident prevent* or error* or medication reconcil* or near miss*)<br>11. (efficiency or economic* or cost* or expenditure* or charge* or fee*1 or (number adj2 appointment*) or (number adj2 admission*) or (number adj2 telephone visit*))<br>12. Waiting Lists/ or Time-to-treatment/ or timeliness<br>13. Health Equity/<br>14. 8 or 9 or 10 or 11 or 12 or 13 15. 7 and 14
**PsycINFO *via* Ovid**	1. (((electronic* or online or on-line or digital*) adj2 (health record* or medical record* or personal record* or patient record*)) or EHR? or EMR? or ephr?)<br>2. (((information or data) adj5 (shar* or exchang*)) or HIE*2 or access*)<br>3. 1 and 2<br>4. (((experience or satisfaction) adj5 (patient* or consumer* or client* or survey or questionnaire*)) or PREM* or patient-reported experience measure*)<br>5. (effectiveness or health outcome*)<br>6. (patient adj2 (safety or harm)) or safety manag* or accident prevent* or error* or medication reconcil* or near miss*<br>7. (efficiency or economic* or cost* or expenditure* or charge* or fee*1 or (number adj2 appointment*) or (number adj2 admission*) or (number adj2 telephone visit*))<br>8. waiting list* or time-to-treatment or timeliness<br>9. equity or health disparities/<br>10. 4 or 5 or 6 or 7 or 8 or 9<br>11. 3 and 10

For peer review only

293 Search themes (facets) and terms derived for each theme relating to the use of EHR and predicted benefits
294 (patient experience, effectiveness and efficiency)

For peer review only

Table 2. Inclusion and exclusion criteria

	**Inclusion criteria**	**Exclusion criteria**
**Population**	Adult subjects (patients and carers)	Individuals 16 years of age and under (e.g. mean age of study sample <16)
**Intervention**	Electronic health record-based interventions, including:<br>• Patient access to EHR<br>• EHR-based reminders / messaging<br>• Unidirectional or bidirectional online patient-provider communication systems (care information exchange platforms);	• Health reminders only
**Comparison**	No intervention (*e.g.* usual care)	
**Outcome**	Any measure related to a) patient-centredness (*e.g.* PREMs), b) effectiveness (*e.g.* health outcomes)*;* c) patient safety (*e.g.* identification of medication discrepancies); d) efficiency (*e.g.* economic evaluation measures and proxies; including service costs, number of consultations/admissions, e) timeliness (*e.g.* waiting lists, time-to-treatment) or f) equity (*e.g.* discrepancies in quality measures between different groups of patients).	Studies that only report cognitive outcomes (*e.g.* intention to), motivational outcomes or other subjective psychological measures
**Study type**	Randomised controlled trials, cluster randomised trials, quasi-experimental, case-control, cohort studies, systematic reviews	

Reference

1. Dimond B. Rights to information access under the Data Protection Act. Br J Nurs. 2005;14(14):774-6.

2. Stange KC. The problem of fragmentation and the need for integrative solutions. Ann Fam Med. 2009;7(2):100-3.

3. Roukema J, Los RK, Bleeker SE, van Ginneken AM, van der Lei J, Moll HA. Paper versus computer: feasibility of an electronic medical record in general pediatrics. Pediatrics. 2006;117(1):15-21.

4. Vest JR. Health information exchange: national and international approaches. Adv Health Care Manag. 2012;12:3-24.

5. House of Parliament. EHR House of Parliament Report. 2016.

6. Delbanco T, Walker J, Bell SK, Darer JD, Elmore JG, Farag N, et al. Inviting patients to read their doctors' notes: a quasi-experimental study and a look ahead. Ann Intern Med. 2012;157(7):461-70.

7. Katz SJ, Nissan N, Moyer CA. Crossing the digital divide: evaluating online communication between patients and their providers. Am J Manag Care. 2004;10(9):593-8.

8. Wright A, Poon EG, Wald J, Feblowitz J, Pang JE, Schnipper JL, et al. Randomized controlled trial of health maintenance reminders provided directly to patients through an electronic PHR. J Gen Intern Med. 2012;27(1):85-92.

9. Nagykaldi Z, Aspy CB, Chou A, Mold JW. Impact of a Wellness Portal on the delivery of patient-centered preventive care. J Am Board Fam Med. 2012;25(2):158-67.

10. Institute of Medicine Committee on Quality of Health Care in America. Crossing the Quality Chasm: A New Health System for the 21st Century. National Academies Press; 2001.

11. Scholl I, Zill JM, Harter M, Dirmaier J. An integrative model of patient-centeredness - a systematic review and concept analysis. PLoS One. 2014;9(9):e107828.

12. Arah OA, Klazinga NS, Delnoij DMJ, ten Asbroek AHA, Custers T. Conceptual frameworks for health systems performance: a quest for effectiveness, quality, and improvement. Int J Qual Health Care. 2003;15(5):377-98.

13. Hussey PS, de Vries H, Romley J, Wang MC, Chen SS, Shekelle PG, et al. A systematic review of health care efficiency measures. Health Serv Res. 2009;44(3):784-805.

14. World Health Organisation. Health Systems: Equity. World Health Organisation; 2017.

15. Wagner KA, Lee FW, Glaser JP. Healthcare information systems: A practical approach for health care management. John Wiley & Sons 2013.

16. Department of Health. The power of information: giving people control of the health and care information they need. Department of Health; 2012.

17. Ross SE, Lin C-T. The effects of promoting patient access to medical records: a review. J Am Med Inform Assoc. 2003;10(2):129-38.

18. Jilka SR, Callahan R, Sevdalis N, Mayer EK, Darzi A. "Nothing About Me Without Me": An Interpretative Review of Patient Accessible Electronic Health Records. J Med Internet Res. 2015;17(6):e161.

19. Davis Giardina T, Menon S, Parrish DE, Sittig DF, Singh H. Patient access to medical records and healthcare outcomes: a systematic review. J Am Med Inform Assoc. 2014;21(4):737-41.

20. de Lusignan S, Mold F, Sheikh A, Majeed A, Wyatt JC, Quinn T, et al. Patients' online access to their electronic health records and linked online services: a systematic interpretative review. BMJ Open. 2014;4(9):e006021.

21. Tenforde M, Nowacki A, Jain A, Hickner J. The association between personal health record use and diabetes quality measures. J Gen Intern Med. 2012;27(4):420-4.

For peer review only

22. McCarrier KP, Ralston JD, Hirsch IB, Lewis G, Martin DP, Zimmerman FJ, et al. Web-based collaborative care for type 1 diabetes: a pilot randomized trial. Diabetes Technol Ther. 2009;11(4):211-7.
23. Grant RW, Wald JS, Schnipper JL, Gandhi TK, Poon EG, Orav EJ, et al. Practice-linked online personal health records for type 2 diabetes mellitus: a randomized controlled trial. Arch Intern Med. 2008;168(16):1776-82.
24. Mold F, de Lusignan S, Sheikh A, Majeed A, Wyatt JC, Quinn T, et al. Patients' online access to their electronic health records and linked online services: a systematic review in primary care. Br J Gen Pract. 2015;65(632):e141-51.
25. Palen TE, Ross C, Powers JD, Xu S. Association of online patient access to clinicians and medical records with use of clinical services. JAMA. 2012;308(19):2012-9.
26. Bergmo TS, Kummervold PE, Gammon D, Dahl LB. Electronic patient-provider communication: will it offset office visits and telephone consultations in primary care? Int J Med Inform. 2005;74(9):705-10.
27. Zhou YY, Garrido T, Chin HL, Wiesenthal AM, Liang LL. Patient access to an electronic health record with secure messaging: impact on primary care utilization. Am J Manag Care. 2007;13(7):418-24.
28. Kummervold PE, Trondsen M, Andreassen H, Gammon D, Hjortdahl P. [Patient-physician interaction over the internet].Erfaringer med lege-pasient-kontakt over Internett. Tidsskr Nor Laegeforen. 2004;124(20):2633-6.
29. Lin C-T, Wittevrongel L, Moore L, Beaty BL, Ross SE. An Internet-based patient-provider communication system: randomized controlled trial. J Med Internet Res. 2005;7(4):e47.
30. Katz SJ, Moyer CA, Cox DT, Stern DT. Effect of a triage-based E-mail system on clinic resource use and patient and physician satisfaction in primary care: a randomized controlled trial. J Gen Intern Med. 2003;18(9):736-44.
31. Moher D, Shamseer L, Clarke M, Ghersi D, Liberati A, Petticrew M, et al. Preferred reporting items for systematic review and meta-analysis protocols (PRISMA-P) 2015 statement. Syst Rev. 2015;4:1.
32. Higgins J, Green S. Cochrane Handbook for Systematic Reviews of Interventions. John Wiley & Sons; 2008.
33. Duval S, Tweedie R. Trim and fill: A simple funnel-plot-based method of testing and adjusting for publication bias in meta-analysis. Biometrics. 2000;56(2):455-63.
34. Guyatt GH, Oxman AD, Vist GE, Kunz R, Falck-Ytter Y, Alonso-Coello P, et al. GRADE: an emerging consensus on rating quality of evidence and strength of recommendations. Bmj. 2008;336(7650):924-6.
35. Silagy CA, Middleton P, Hopewell S. Publishing protocols of systematic reviews: comparing what was done to what was planned. JAMA 2002;287(21):2831-4.

For peer review only

For peer review only - http://bmjopen.bmj.com/site/about/guidelines.xhtml

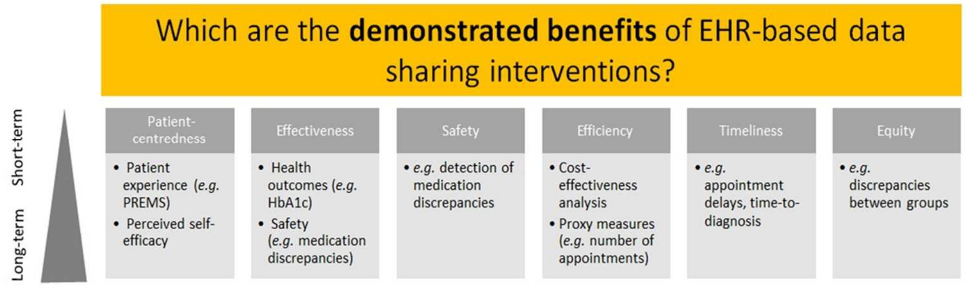

Figure 1. Conceptual model

338x190mm (96 x 96 DPI)

review only

PRISMA-P (Preferred Reporting Items for Systematic review and Meta-Analysis Protocols) 2015 checklist: recommended items to address in a systematic review protocol*

Section and topic	Item No	Checklist item	Page
**ADMINISTRATIVE INFORMATION**			
Title:			
Identification	1a	Identify the report as a protocol of a systematic review	1, 2, 8, 9, 10
Update	1b	If the protocol is for an update of a previous systematic review, identify as such	Not applicable
Registration	2	If registered, provide the name of the registry (such as PROSPERO) and registration number	2
Authors:			
Contact	3a	Provide name, institutional affiliation, e-mail address of all protocol authors; provide physical mailing address of corresponding author	1
Contributions	3b	Describe contributions of protocol authors and identify the guarantor of the review	10
Amendments	4	If the protocol represents an amendment of a previously completed or published protocol, identify as such and list changes; otherwise, state plan for documenting important protocol amendments	Not applicable
Support:			
Sources	5a	Indicate sources of financial or other support for the review	10
Sponsor	5b	Provide name for the review funder and/or sponsor	10
Role of sponsor or funder	5c	Describe roles of funder(s), sponsor(s), and/or institution(s), if any, in developing the protocol	10
**INTRODUCTION**			
Rationale	6	Describe the rationale for the review in the context of what is already known	3
Objectives	7	Provide an explicit statement of the question(s) the review will address with reference to participants, interventions, comparators, and outcomes (PICO)	6
**METHODS**			
Eligibility criteria	8	Specify the study characteristics (such as PICO, study design, setting, time frame) and report characteristics (such as years considered, language, publication status) to be used as criteria for eligibility for the review	6,14
Information sources	9	Describe all intended information sources (such as electronic databases, contact with study authors, trial registers or other grey literature sources) with planned dates of coverage	6
Search strategy	10	Present draft of search strategy to be used for at least one electronic database, including planned limits, such that it could be repeated	6, 12-16

For peer review only

Study records:			
Data management	11a	Describe the mechanism(s) that will be used to manage records and data throughout the review	7
Selection process	11b	State the process that will be used for selecting studies (such as two independent reviewers) through each phase of the review (that is, screening, eligibility and inclusion in meta-analysis)	7
Data collection process	11c	Describe planned method of extracting data from reports (such as piloting forms, done independently, in duplicate), any processes for obtaining and confirming data from investigators	7
Data items	12	List and define all variables for which data will be sought (such as PICO items, funding sources), any pre-planned data assumptions and simplifications	7
Outcomes and prioritization	13	List and define all outcomes for which data will be sought, including prioritization of main and additional outcomes, with rationale	6
Risk of bias in individual studies	14	Describe anticipated methods for assessing risk of bias of individual studies, including whether this will be done at the outcome or study level, or both; state how this information will be used in data synthesis	6
Data synthesis	15a	Describe criteria under which study data will be quantitatively synthesised	6
	15b	If data are appropriate for quantitative synthesis, describe planned summary measures, methods of handling data and methods of combining data from studies, including any planned exploration of consistency (such as I^2, Kendall's τ)	7
	15c	Describe any proposed additional analyses (such as sensitivity or subgroup analyses, meta-regression)	7
	15d	If quantitative synthesis is not appropriate, describe the type of summary planned	7
Meta-bias(es)	16	Specify any planned assessment of meta-bias(es) (such as publication bias across studies, selective reporting within studies)	7
Confidence in cumulative evidence	17	Describe how the strength of the body of evidence will be assessed (such as GRADE)	7

*** It is strongly recommended that this checklist be read in conjunction with the PRISMA-P Explanation and Elaboration (cite when available) for important clarification on the items. Amendments to a review protocol should be tracked and dated. The copyright for PRISMA-P (including checklist) is held by the PRISMA-P Group and is distributed under a Creative Commons Attribution Licence 4.0.**

From: Shamseer L, Moher D, Clarke M, Ghersi D, Liberati A, Petticrew M, Shekelle P, Stewart L, PRISMA-P Group. Preferred reporting items for systematic review and meta-analysis protocols (PRISMA-P) 2015: elaboration and explanation. BMJ. 2015 Jan 2;349(jan02 1):g7647.

For peer review only

BMJ Open

Impact of sharing electronic health records with patients on the quality and safety of care: a systematic review and narrative synthesis protocol

Journal:	*BMJ Open*
Manuscript ID	bmjopen-2017-020387.R1
Article Type:	Protocol
Date Submitted by the Author:	26-Mar-2018

Complete List of Authors:	Neves, Ana Luisa; Imperial College London, Center for Health Policy, Institute of Global Health Innovation<br>Carter, Alexander; Imperial College London, Center for Health Policy, Institute of Global Health Innovation<br>Freise, Lisa; Imperial College London, Center for Health Policy, Institute of Global Health Innovation<br>Laranjo, Liliana; Macquarie University Faculty of Science, Australian Institute of Health Innovation; Universidade Nova de Lisboa, Centro de Investigação em Saúde Pública, Escola Nacional de Saúde Pública<br>Darzi, Ara; Imperial College London, Center for Health Policy, Institute of Global Health Innovation<br>Mayer, Erik; Imperial College London, Center for Health Policy, Institute of Global Health Innovation

**Primary Subject Heading**:	Health informatics
Secondary Subject Heading:	Evidence based practice, Health policy
Keywords:	Health informatics < BIOTECHNOLOGY & BIOINFORMATICS, Information technology < BIOTECHNOLOGY & BIOINFORMATICS, Quality in health care < HEALTH SERVICES ADMINISTRATION & MANAGEMENT, Health policy < HEALTH SERVICES ADMINISTRATION & MANAGEMENT, Health & safety < HEALTH SERVICES ADMINISTRATION & MANAGEMENT

SCHOLARONE™
Manuscripts

Impact of sharing electronic health records with patients on the quality and safety of care: a systematic review and narrative synthesis protocol

Authors: Ana Luisa Neves[1] (ALN) - ana.luisa.neves14@imperial.ac.uk, Alexander Carter (AC) - a.w.carter@imperial.ac.uk, Lisa Freise[1] (LF) - l.freise16@imperial.ac.uk, Liliana Laranjo[2] - liliana.laranjo@mq.edu.au, Ara Darzi[1] (AD) - a.darzi@imperial.ac.uk, Erik Mayer[1] (EM) - e.mayer@imperial.ac.uk

[1]Center for Health Policy, Institute of Global Health Innovation, Imperial College London, London, UK
[2]Australian Institute of Health Innovation, Centre for Health Informatics, Macquarie University, Sidney, Australia

Corresponding author

Name: Ana Luisa Neves

Address: Centre for Health Policy, Room 1035/7, QEQM Wing, St Mary's Campus, London W2 1NY

E-mail: ana.luisa.neves14@ic.ac.uk

Telephone: 02033127259

Fax number: 02033126309

Keywords [MeSH terms]: Electronic Health Records, Patient Access to Records, Patient-Centered Care, Quality of Health Care, Patient Safety

Word count: 2145 words

For peer review only

Abstract

Introduction: Providing patients with access to Electronic Health Records (EHR) has emerged as a promising solution to improve quality of care and safety. However, there is a considerable gap between the predicted and demonstrated impact of these interventions. As the efforts to develop and implement EHR-based data sharing platforms mature and scale up worldwide, there is a need to evaluate the impact of these interventions and to weigh their relative risks and benefits, in order to inform evidence-based health policies. The aim of this work is to systematically characterise and appraise the demonstrated benefits and risks of sharing EHR with patients, by mapping them across the six domains of quality of care of the Institute of Medicine (IOM) analytical framework (*i.e.* patient-centredness, effectiveness, efficiency, timeliness, equity and safety).

Methods and analysis: CINAHL, Cochrane, Embase, HMIC, Medline/PubMed and PsycINFO databases will be searched from January 1997 to August 2017. Primary outcomes will include measures related with the six domains of quality of care of the IOM analytical framework (*i.e.* patient-centredness, effectiveness, efficiency, timeliness, equity and safety). Valid studies will be assessed for their quality by two independent researchers, using the Cochrane Risk of Bias Tool, the ROBINS-I tool and the Drummond's checklist. A narrative synthesis will be conducted for all included studies. Subgroup analysis will be performed by domain of quality of care domain and by time scale (*i.e.* short-, medium- or long-term impact). The body of evidence will be summarised in a Summary of Findings table and the strength of the body of evidence will be assessed according to the GRADE criteria. This protocol was registered in PROSPERO (CRD42017070092).

Ethics and dissemination: Findings will be disseminated widely through peer-reviewed publication and conference presentations.

Strengths and limitations of this study

- Comprehensive characterisation of interventions sharing EHR with patients
- Summary and appraisal of existing evidence on the potential benefits and risks of these interventions, grouped by domain of quality of care
- Map the contribution of these interventions in short-, medium- and long-term time frames, in order to customise informed decisions in health policies
- Expected limitations include the heterogenous nature of the outcomes assessed and the potentially reduced sample size in subgroup analyses, which may negatively impact the statistical power in data synthesis.

For peer review only

Introduction

Although, in England, patients have had the legal right to access their health records since 1998, access to paper-based health records is mediated by health professionals and data controllers, through a cumbersome procedural process [1]. Additionally, as health information is fragmented between different organisations and levels of care, data access requests are often unable to provide a comprehensive health history record [2, 3].

In the last decade, electronic health records (EHR), have emerged as a promising solution to enhance patients' access to centralised medical information [4]. The adoption of EHR by primary care practices, hospitals and other healthcare organizations has steadily increased in the last years. In England, the percentage of General Practice surgeries that allowed patients to access their medical records online increased from 3% to 97% between April 2014 and February 2016 [5]. Patients' willingness and ability to access their health information through web portals is influenced by both individual (*i.e.* age, ethnicity, education level, health literacy and health status) and by health care delivery factors (*i.e.* provider endorsement and portal usability) [6, 7]. Various EHR-based platforms are currently used to share health information with patients, including direct online access, with or without patient-provider communication systems [8, 9] and health maintenance reminders [10, 11]. As these efforts mature and scale up worldwide, there is a need to evaluate the impact of interventions sharing EHR with patients, in order to weigh their relative risks and benefits and inform evidence-based health policies.

The Institute of Medicine (IOM) identified six domains of health care quality: patient-centredness, effectiveness, efficiency, timeliness, equity and safety [12]. Patient-centredness ensures that the care provided respects and responds to individual patient preferences, needs and values, thus incorporating these in clinical decisions [12, 13]. Health care shall provide evidence-based services, which can be ultimately expressed as improvements in health outcomes (*i.e.* effectiveness) [14], while ensuring patient safety (*i.e.* prevention of errors and adverse effects associated with health care) [12]. Other aspects of quality of delivery of care include the minimisation of the waste of human, physical or economical resources (*i.e.* efficiency), the reduction of waits and harmful delays (*i.e.* timeliness) and the reduction of avoidable differences on the delivery of care between different groups of health care users (*i.e.* equity) [12, 15, 16].

Providing patients with access to their health records has been linked to theorised benefits in four major domains of health care quality: patient-centredness, effectiveness, safety and efficiency [17-19]. However, despite the growing body of evidence on the theorised benefits of sharing EHR with patients on

For peer review only

these domains, there is still a considerable gap between the predicted and demonstrated benefits of these interventions [20].

In order to analyse the effect of providing patients access to their medical records on quality outcomes, Davis-Giardina T. *et al* performed a systematic review including studies published between 1970 and 2012 [21]. According to this work, a limited amount of evidence suggests that access to medical records improves patient satisfaction and enhances patient-provider communication [21]. Similarly, a systematic review from de Lusignan *et al.* reported that providing patients online access to their EHR increased convenience and satisfaction [22]. These findings are in line with the model proposed by Otte-Trojel T. *et al*, according to which sharing EHR with patients can improve both patient-provider communication and patient satisfaction, by increasing continuity of care and patient convenience, respectively [23].

Conversely, no clear benefits were found on effectiveness [21]. Until 2012, only a few studies evaluated the impact on effectiveness studies, most focusing on type 2 diabetes and with inconsistent results. Tenforde *et al* showed that providing access to medical records was associated with lower glycated haemoglobin A1c values [24]; however, no significant effect was found in three other studies assessing diabetes-specific effectiveness measures [25, 26]. One of the limitations of this review consists in the inclusion of studies evaluating the impact of sharing both electronic and paper-based health records - and this heterogeneity might mask potential specific benefits and risks of sharing EHR with patients. Furthermore, as pointed by the authors, the paucity of papers published up to that date resulted in a tendency to include small and methodologically less robust studies, thus increasing the risk of selective reporting and/or publication bias [21]. Mold F. *et al* also performed a systematic review assessing the impact of providing patients with access to their EHR; based in studies published between 1999 and 2012, this work found a positive influence in patient safety [27].

However, the authors were unable to find a consistent beneficial effect on efficiency measures (*i.e.* number of face-to-face visits and telephone appointments) in both reviews [21, 27]. While some studies reported an increase in the number of face-to-face consultations [8, 28] , others document a decrease [11, 29, 30]. Similarly, inconsistent results were found regarding the impact on telephone consultations: only one study reported a decline in the total number [31], whilst six other studies reported either no change or an increase [9, 28-30, 32, 33]. It is important to note, however, that most of the included studies assessing efficiency measures included in this review showed a high risk of bias, mostly related to either unclear or absent blinding methods [27].

The landmark reviews of Mold F. *et al* [27], Davis-Giardina T. *et al* [21], Ammenwerth E. *et al* [34] and Goldweig CL *et al* [7] provide a comprehensive characterisation of the literature published until 2013,

For peer review only

highlighting the paucity and the scientific limitations of the evidence published until that date. Although these reviews were unable to demonstrate clear benefits on efficiency and effectiveness measures, the debates around patients' rights and data ownership in the digital era, and the need to improve patient-centredness of health care delivery have acted as strong drivers to allocate resources to interventions and platforms aiming to share EHR with patients. As consequence of these efforts, it is plausible that studies performed in the last 5 years can provide further clarification for this evidence gap.

Furthermore, systematic reviews performed to the date do not address all domains of quality of care; in particular, the impact of sharing EHR with patients on timeliness or equity has not been addressed [7, 21, 27, 34, 35]. This is a particularly relevant gap in knowledge, given that interventions aimed at improving the quality of care do not necessarily improve all specific domains, and may even have a deleterious effect in some of them.

This review will expand on the above-mentioned work, in order to identify recent methodological and scientific progress until June 2017. Following the PRISMA-P checklist as guidance [36], we propose a systematic and reproducible strategy to query the literature on the demonstrated benefits and risks of sharing EHR with patients and map these results in a comprehensive framework of health care quality measures.

Research aims

The main objectives of this review are: 1) to systematically characterise interventions sharing EHR with patients; and 2) to assess the demonstrated risks and benefits of these interventions on patient-centredness, effectiveness, safety, efficiency, timeliness and equity, compared to usual care (no intervention). As secondary aim, we will map the contribution of these interventions in short-, medium- and long-term timeframes (Figure 1).

Methods and analysis

Search strategy

The search strategy will be performed using resources that enhance methodological transparency and improve the reproducibility of the results and evidence synthesis. A search of the literature from the last 20 years (January 1997 – August 2017) will be performed on CINAHL, Cochrane, Embase, HMIC, Medline/PubMed and PsycINFO. Search strings (Table 1) will combine free terms and controlled

For peer review only

vocabulary, whenever supported. We will also search grey literature sources, including registrations in the International Prospective Register of Systematic Reviews (PROSPERO), reports of relevant stakeholder organisations (NHS Digital, AMIA, eHealth at WHO, International Society for Telemedicine and eHealth), and conference proceedings (last 5 years) of several related conferences (American Medical Informatics Association [AMIA], MedInfo, Medicine 2.0, Medicine X), in order to identify possible additional studies that meet the inclusion criteria. Language restrictions will be applied and only articles in English will be included.

Study selection criteria

A summary of the participants, interventions, comparators and outcomes (PICO) considered, as well as the type of studies included, is provided in Table 2.

The systematic review will focus on studies on adult subjects, including both patients and carers (mean age of study sample ≥ 16 years). The systematic review does not focus on a particular disease area or health system setting as it intends to comprehensively characterise the scope of interventions sharing EHR with patients.

Studies assessing the impact of sharing EHR with patients, either isolated or as part of a multicomponent intervention, will be included. Included interventions will comprise: 1) web-based patient access to EHR; 2) EHR-based health reminders / messaging or 3) online patient-provider communication systems (health information exchange platforms). Studies focusing on health reminders only (not EHR-based) or appointment reminders will not be considered. The comparator will be 'no intervention' (*e.g.* usual care).

Primary outcomes will include any measure related to a) patient-centredness (*e.g.* patient-reported experience measures (PREMs), b) effectiveness (*e.g.* health outcomes); c) patient safety (*e.g.* identification of medication discrepancies); d) efficiency (*e.g.* economic evaluation measures and proxies; including service costs, number of consultations/admissions, e) timeliness (*e.g.* waiting lists, time-to-treatment) and f) equity (*e.g.* discrepancies in quality measures between different groups of patients) (Figure 1). Studies that only report cognitive outcomes (*e.g.* intent), motivational outcomes or other subjective psychological measures will be excluded. The types of study considered in this systematic review will be a) randomised controlled trials; b) cluster randomised trials; c) quasi-experimental studies; d) case-control studies, e) cohort studies and f) cost-effectiveness studies. The reference lists of systematic reviews identified in this search will also be screened to ensure all eligible studies are captured.

For peer review only

Screening and data extraction

Quantitative studies will be independently assessed by three reviewers and reported using the Preferred Reporting Items for Systematic Reviews and Meta-Analyses (PRISMA-P) flow diagram [36]. Initial screening of studies will be based on the information contained in their titles and abstracts and will be conducted by two independent investigators. Full-paper screening will be conducted by the same independent investigators. Cohen's kappa will be used to measure inter-coder agreement in each screening phase. When there are doubts regarding inclusion or exclusion, a third investigator will be involved in the decision. Two independent investigators will extract information from the included studies into a standardized form. The data collected for each study will include: name of the first author, year of publication, technology, intervention components and characteristics, study duration, participants' and setting characteristics, outcomes and retention rates. Two investigators will review the abstraction form for consistency. Disagreements will be resolved by a third investigator.

Quality assessment

The quality of randomised controlled trials and cluster randomized trials will be assessed using the Cochrane Risk of Bias Tool [37], that assesses the following study-level aspects: a) randomisation sequence allocation; b) allocation concealment; c) blinding; d) completeness of outcome data and e) selective outcome reporting. The quality of non-randomised intervention studies (*i.e.* case control, cohort, quasi-experimental) will be appraised using the ROBINS-I tool, which assesses bias due to a) confounding, b) selection of participants, c) classification of interventions, d) deviations from intended interventions, e) missing data, f) measurement of outcomes and g) selection of reported results [38]. For cost-effectiveness studies, the Drummond's checklist for assessing economic evaluations will be used [39]. Two independent reviewers will score the selected studies and disagreements will be resolved by a third person.

The risk of bias for each outcome across individual studies will be summarised as a narrative statement, and supported by a risk of bias table. A review-level narrative summary of the risk of bias will also be provided.

For peer review only

Descriptive analysis and meta-analysis

Planned subgroup analysis will be performed by domain of quality of care (IOM framework) and by time scale (*i.e.* short-, medium- or long-term impact). For studies with a high or unclear risk of bias, defined as high or unclear risk in 50% or more of the quality assessment outcomes, a narrative description of the risk of bias will be provided. Risk of bias assessments will be incorporated into synthesis by performing sensitivity analysis (*i.e.* limiting to studies at lowest risk of bias in a secondary analysis). Depending on the amount of information retrieved, subgroup analysis will also be performed for specific diseases.

A narrative synthesis will be conducted for all the included studies. Parallel-group trials that are deemed comparable in relevant ways will be pooled together for a summary effect. Whenever possible, continuous and dichotomous outcomes will be pooled together for meta-analysis purposes. All effect sizes will be transformed into a common metric, in order to make them comparable across studies — the bias-corrected standardized difference in means (Hedges' g) — classified as positive when in favor of the intervention and negative when in favor of the control. Heterogeneity will be assessed using I^2. The presence of publication bias will be evaluated by use of a funnel plot and the Duval and Tweedie's trim and fill method [40].

The body of evidence will be summarised in a Summary of Findings table and the strength of the body of evidence will be assessed according to GRADE criteria [41].

Patient and public involvement

Our research question emerged from the implementation evaluation of the Care Information Exchange (CIE), a pilot web portal/patient-controlled EHR happening across a 2.4 million population in North West London. CIE implementation evaluation was shaped by its steering group, which included lay partners, and their perspectives reinforced that our research question was relevant and aligned with patients' interest.

Patients were not directly involved in the design of this study. As this is a protocol for a systematic review and no participant recruitment will take place, their involvement on the recruitment and dissemination of findings to participants was not applicable.

However, patient partners will be included in the interpretation of our results, in the co-development of a dissemination strategy, and in summarising the research findings into lay summaries and reports, in order to raise awareness and stimulate public participation on this topic.

For peer review only

276 *Amendments*

277 Any amendments to this protocol will be documented with reference to saved searches and analysis
278 methods, which will be recorded in bibliographic databases (Ovid), Endnote, and Excel templates for data
279 collection and synthesis.

280

For peer review only

Discussion

As the implementation of interventions to share EHR with patients scales up worldwide, the systematic evaluation of their impact emerges as a priority research topic.

One of the strengths of the proposed study is to apply a reproducible and transparent procedure for systematic review of the literature. In this protocol, we clearly describe the types of studies, participants, interventions, and outcomes that will be included, as well as the data sources, search strategy, data extraction methods (including quality assessment) and methods of combining data [42]. By publishing the research protocol, we reinforce the clarity of the strategy and minimise the risk of bias, namely selective outcome reporting [37]. Second, we will focus solely on the impact of EHR-based studies, increasing the sensitivity to detect specific benefits of this type of intervention. Third, for the first time, we aim to comprehensively evaluate both the benefits and risks of these interventions in a wide range of domains of quality of care, as defined by the IOM, and in diverse time frames. This results shall provide high-level information to inform, support and customise decisions in health policies.

Potential limitations of this study include the heterogeneity of measures and outcomes evaluated and the potentially reduced number of studies in subgroup analyses, which may negatively influence the statistical power in data synthesis.

For peer review only

Acknowledgements

We thank Jacqueline Cousins (Library Manager and Liaison Librarian at Imperial College London) for the support improving the composition of the search terms and procedural aspects of the search strategy. We would also like to acknowledge Dr Søren Rud Kristensen (Senior Lecturer in Health Economics at the Centre for Health Policy at the Institute of Global Health Innovation) and Dr Joachim Marti (Senior Lecturer in Health Economics at the Centre for Health Policy at the Institute of Global Health Innovation) for their advices regarding the quality appraisal of cost-effectiveness studies, and Miss Anna Lawrence-Jones (Patient and Public Involvement and Engagement Manager at Imperial National Institute for Health Research (NIHR) Patient Safety Translation Research Centre) for the useful discussions around public and patient involvement.

Ethics and dissemination

Not applicable.

Authors' contributions

ALN, AC and EKM conceptualised this research. ALN, AC and LL designed the protocol. ALN and LF defined the concepts and search items. EKM and AD contributed to the conceptualisation and commented on the multiple versions of the protocol. The manuscript was written by ALN with contributions from all authors.

Funding statement

This work is supported by the Sowerby Program and by the National Institute for Health Research (NIHR) Patient Safety Translation Research Centre. The views of the authors do not necessarily reflect those of the NHS, NIHR or the Department of Health.

Competing interests' statement

We declare no conflict(s) of interest associated with this research.

For peer review only

Data sharing statement

No additional data relevant to this protocol are available for distribution. All relevant data and search terms are published in this manuscript.

For peer review only

Table 1. Concepts and search items

Database	Search items
**CINAHL *via* EBSCO**	1. (((electronic* or online or on-line or digital*) N1 (health record* or medical record* or personal record* or patient record*)) or EHR# or EMR# or ephr#)<br>2. ((information or data) N4 (shar* or exchang*)) or HIE or HIEs or access*)<br>3. #1 and #2<br>4. (((experience or satisfaction) N4 (patient* or consumer* or client* or survey or questionnaire*)) or PREM* or patient-reported experience measure*)<br>5.(effectiveness or health outcome*)<br>6. (patient N1 (safety or harm)) or safety manag* or accident prevent* or error* or medication reconcil* or near miss*<br>7. (efficiency or economic* or cost* or expenditure* or charge* or fee*1 or (number N1 appointment*) or (number adj2 admission*) or (number N1 telephone visit*))<br>8. waiting lists or timeliness or time-to-treatment<br>9. health equity<br>10. #4 or #5 or #6 or #7 or #8 or #9<br>13. 3 and 10
**Cochrane *via* url:** http://onlinelibrary.wiley.com/cochranelibrary/search/advanced	1. (((electronic* or online or on-line or digital*) near/1 (health record* or medical record* or personal record* or patient record*)) or EHR or EHRs or EMR or EMRs or ephr or ephrs)<br>2. Electronic Health Records [MesH]<br>3. #1 or #2<br>4. (((information or data) near/4 (shar* or exchang*)) or HIE or HIES or access*)<br>5. Information Dissemination [MesH]<br>6. #4 or #5<br>7. #3 and #6<br>8. (((experience or satisfaction) near/4 (patient* or consumer* or client* or survey or questionnaire*)) or PREM* or patient-reported experience measure*)<br>9. (effectiveness or health outcome*)<br>10. (patient near/1 safety) or (patient near/1 harm) or safety manag* or accident prevent* or error* or medication reconcil*<br>11. (efficiency or economic* or cost* or expenditure* or charge* or fee* or (number near/1 appointment*) or (number near/1 admission*) or (number near/1 telephone visit*))<br>12. time-to-treatment or timeliness<br>13. waiting lists [MesH]<br>14. health equity [MesH]<br>15. #8 or #9 or #10 or #11 or #12 or #13 or #14

For peer review only

	17. 7 and 15
**Embase *via* Ovid**	1. (((electronic* or online or on-line or digital*) adj2 (health record* or medical record* or personal record* or patient record*)) or EHR? or EMR? or ephr?)<br>2. Electronic health record/<br>3. 1 or 2<br>4. (((information or data) adj5 (shar* or exchang*)) or HIE*2 or access*)<br>5. Information dissemination/<br>6. 4 or 5<br>7. 3 and 6<br>8. (((experience or satisfaction) adj5 (patient* or consumer* or client* or survey or questionnaire*)) or PREM* or patient-reported experience measure*)<br>9. (effectiveness or health outcome*) 10. (patient adj2 (safety or harm)) or safety manag* or accident prevent* or error* or medication reconcil* or near miss*<br>11. (efficiency or economic* or cost* or expenditure* or charge* or fee*1 or (number adj2 appointment*) or (number adj2 admission*) or (number adj2 telephone visit*))<br>12. waiting list* or time to treatment/ or timeliness<br>13. health equity/<br>14. 8 or 9 or 10 11 or 12 or 13<br>15. 7 and 14
**HMIC *via* Ovid**	1. (((electronic* or online or on-line or digital*) adj2 (health record* or medical record* or personal record* or patient record*)) or EHR? or EMR? or ephr?)<br>2. Electronic patient records/<br>3. 1 or 2<br>4. (((information or data) adj5 (shar* or exchang*)) or HIE*2 or access*)<br>5. Information exchange/<br>6. 4 or 5<br>7. 3 and 6<br>8. (((experience or satisfaction) adj5 (patient* or consumer* or client* or survey or questionnaire*)) or PREM* or patient-reported experience measure*)<br>9. (effectiveness or health outcome*) 10. (patient adj2 (safety or harm)) or safety manag* or accident prevent* or error* or medication reconcil* or near miss*<br>11. (efficiency or economic* or cost* or expenditure* or charge* or fee*1 or (number adj2 appointment*) or (number adj2 admission*) or (number adj2 telephone visit*))<br>12. waiting lists/ or patient waiting time or timeliness<br>13. health inequalities/ or equity

For peer review only

	14. 8 or 9 or 10 or 11 or 12 or 13<br>15. 7 and 14
**Medline via Ovid**	1. (((electronic* or online or on-line or digital*) adj2 (health record* or medical record* or personal record* or patient record*)) or EHR? or EMR? or ephr?)<br>2. Electronic Health Records/<br>3. 1 or 2<br>4. (((information or data) adj5 (shar* or exchang*)) or HIE*2 or access*<br>5. Information Dissemination/<br>6. 4 or 5<br>7. 3 and 6<br>8. (((experience or satisfaction) adj5 (patient* or consumer* or client* or survey or questionnaire*)) or PREM* or patient-reported experience measure*)<br>9. (effectiveness or health outcome*)<br>10. ((patient adj2 (safety or harm)) or safety manag* or accident prevent* or error* or medication reconcil* or near miss*)<br>11. (efficiency or economic* or cost* or expenditure* or charge* or fee*1 or (number adj2 appointment*) or (number adj2 admission*) or (number adj2 telephone visit*))<br>12. Waiting Lists/ or Time-to-treatment/ or timeliness<br>13. Health Equity/<br>14. 8 or 9 or 10 or 11 or 12 or 13 15. 7 and 14
**PsycINFO *via* Ovid**	1. (((electronic* or online or on-line or digital*) adj2 (health record* or medical record* or personal record* or patient record*)) or EHR? or EMR? or ephr?)<br>2. (((information or data) adj5 (shar* or exchang*)) or HIE*2 or access*)<br>3. 1 and 2<br>4. (((experience or satisfaction) adj5 (patient* or consumer* or client* or survey or questionnaire*)) or PREM* or patient-reported experience measure*)<br>5. (effectiveness or health outcome*)<br>6. (patient adj2 (safety or harm)) or safety manag* or accident prevent* or error* or medication reconcil* or near miss*<br>7. (efficiency or economic* or cost* or expenditure* or charge* or fee*1 or (number adj2 appointment*) or (number adj2 admission*) or (number adj2 telephone visit*))<br>8. waiting list* or time-to-treatment or timeliness<br>9. equity or health disparities/<br>10. 4 or 5 or 6 or 7 or 8 or 9<br>11. 3 and 10

Search themes (facets) and terms derived for each theme relating to the use of EHR and predicted benefits (patient experience, effectiveness and efficiency)

For peer review only

Table 2. Inclusion and exclusion criteria

	Inclusion criteria	Exclusion criteria
**Population**	Adult subjects (patients and carers)	Individuals 16 years of age and under (e.g. mean age of study sample <16)
**Intervention**	Electronic health record-based interventions, including:<br>• Patient access to EHR<br>• EHR-based reminders / messaging<br>• Unidirectional or bidirectional online patient-provider communication systems (care information exchange platforms);	• Health reminders only
**Comparison**	No intervention (*e.g.* usual care)	
**Outcome**	Any measure related to a) patient-centredness (*e.g.* PREMs), b) effectiveness (*e.g.* health outcomes); c) patient safety (*e.g.* identification of medication discrepancies); d) efficiency (*e.g.* economic evaluation measures and proxies; including service costs, number of consultations/admissions, e) timeliness (*e.g.* waiting lists, time-to-treatment) or f) equity (*e.g.* discrepancies in quality measures between different groups of patients).	Studies that only report cognitive outcomes (*e.g.* intention to), motivational outcomes or other subjective psychological measures
**Study type**	Randomised controlled trials, cluster randomised trials, quasi-experimental, case-control, cohort studies, cost-effectiveness	

For peer review only

Reference

1. Dimond, B., *Rights to information access under the Data Protection Act.* British Journal of Nursing, 2005. **14**(14): p. 774-6.
2. Stange, K.C., *The problem of fragmentation and the need for integrative solutions.* Annals of Family Medicine, 2009. **7**(2): p. 100-3.
3. Roukema, J., et al., *Paper versus computer: feasibility of an electronic medical record in general pediatrics.* Pediatrics, 2006. **117**(1): p. 15-21.
4. Vest, J.R., *Health information exchange: national and international approaches.* Advances in Health Care Management, 2012. **12**: p. 3-24.
5. Houses of Parliament, *EHR House of Parliament Report.* 2016.
6. Irizarry, T., A. DeVito Dabbs, and C.R. Curran, *Patient Portals and Patient Engagement: A State of the Science Review.* Journal of Medical Internet Research, 2015. **17**(6): p. e148.
7. Goldzweig, C.L., et al., *Electronic patient portals: evidence on health outcomes, satisfaction, efficiency, and attitudes: a systematic review.* Annals of Internal Medicine, 2013. **159**(10): p. 677-87.
8. Delbanco, T., et al., *Inviting patients to read their doctors' notes: a quasi-experimental study and a look ahead.* Annals of Internal Medicine, 2012. **157**(7): p. 461-70.
9. Katz, S.J., N. Nissan, and C.A. Moyer, *Crossing the digital divide: evaluating online communication between patients and their providers.* The American Journal of Managed Care, 2004. **10**(9): p. 593-8.
10. Wright, A., et al., *Randomized controlled trial of health maintenance reminders provided directly to patients through an electronic PHR.* Journal of General Internal Medicine, 2012. **27**(1): p. 85-92.
11. Nagykaldi, Z., et al., *Impact of a Wellness Portal on the delivery of patient-centered preventive care.* Journal of the American Board of Family Medicine, 2012. **25**(2): p. 158-67.
12. America, I.o.M.C.o.Q.o.H.C.i., *Crossing the Quality Chasm: A New Health System for the 21st Century*. 2001, Washington (DC): National Academies Press.
13. Scholl, I., et al., *An integrative model of patient-centeredness - a systematic review and concept analysis.* PloS One, 2014. **9**(9): p. e107828.
14. Arah, O.A., et al., *Conceptual frameworks for health systems performance: a quest for effectiveness, quality, and improvement.* Journal of the International Society for Quality in Health Care, 2003. **15**(5): p. 377-98.
15. Hussey, P.S., et al., *A systematic review of health care efficiency measures.* Health services Research, 2009. **44**(3): p. 784-805.
16. World Health Organisation, *Health Systems: Equity, 2017.* Available at http://www.who.int/healthsystems/topics/equity/en/. Last access: 26th March 2018.
17. Wagner, K.A., F.W. Lee, and J.P. Glaser, *Healthcare information systems: A practical approach for health care management.*, J.W. Sons. 2013.
18. Department of Health, *The power of information: giving people control of the health and care information they need,* 2012. Available at: https://www.gov.uk/government/publications/giving-people-control-of-the-health-and-care-information-they-need. Last access: 26th March 2018.
19. Ross, S.E. and C.-T. Lin, *The effects of promoting patient access to medical records: a review.* Journal of the American Medical Informatics Association, 2003. **10**(2): p. 129-38.
20. Jilka, S.R., et al., *"Nothing About Me Without Me": An Interpretative Review of Patient Accessible Electronic Health Records.* Journal of Medical Internet Research, 2015. **17**(6): p. e161.

For peer review only

21. Davis Giardina, T., et al., *Patient access to medical records and healthcare outcomes: a systematic review.* Journal of the American Medical Informatics Association, 2014. **21**(4): p. 737-41.
22. de Lusignan, S., et al., *Patients' online access to their electronic health records and linked online services: a systematic interpretative review.* BMJ Open, 2014. **4**(9): p. e006021.
23. Otte-Trojel, T., et al., *How outcomes are achieved through patient portals: a realist review.* Journal of the American Medical Informatics Association, 2014. **21**(4): p. 751-7.
24. Tenforde, M., et al., *The association between personal health record use and diabetes quality measures.* Journal of General Internal Medicine, 2012. **27**(4): p. 420-4.
25. McCarrier, K.P., et al., *Web-based collaborative care for type 1 diabetes: a pilot randomized trial.* Diabetes Technology & Therapeutics, 2009. **11**(4): p. 211-7.
26. Grant, R.W., et al., *Practice-linked online personal health records for type 2 diabetes mellitus: a randomized controlled trial.* Archives of Internal Medicine, 2008. **168**(16): p. 1776-82.
27. Mold, F., et al., *Patients' online access to their electronic health records and linked online services: a systematic review in primary care.* The British Journal of General Practice, 2015. **65**(632): p. e141-51.
28. Palen, T.E., et al., *Association of online patient access to clinicians and medical records with use of clinical services.* Journal of the American Medical Association, 2012. **308**(19): p. 2012-9.
29. Bergmo, T.S., et al., *Electronic patient-provider communication: will it offset office visits and telephone consultations in primary care?* International Journal of Medical Informatics, 2005. **74**(9): p. 705-10.
30. Zhou, Y.Y., et al., *Patient access to an electronic health record with secure messaging: impact on primary care utilization.* The American Journal of Managed Care, 2007. **13**(7): p. 418-24.
31. Kummervold, P.E., et al., *[Patient-physician interaction over the internet].* Tidsskr Nor Laegeforen. 2004 Oct 21;124(20):2633-6.
32. Lin, C.-T., et al., *An Internet-based patient-provider communication system: randomized controlled trial.* Journal of Medical Internet Research, 2005. **7**(4): p. e47.
33. Katz, S.J., et al., *Effect of a triage-based E-mail system on clinic resource use and patient and physician satisfaction in primary care: a randomized controlled trial.* Journal of General Internal Medicine, 2003. **18**(9): p. 736-44.
34. Ammenwerth, E., P. Schnell-Inderst, and A. Hoerbst, *The impact of electronic patient portals on patient care: a systematic review of controlled trials.* Journal of Medical Internet research, 2012. **14**(6): p. e162.
35. Ammenwerth, E., et al., *Adult patient access to electronic health records (Protocol).* Cochrane Database of Systematic Reviews 2017(6): p. CD012707.
36. Moher, D., et al., *Preferred reporting items for systematic review and meta-analysis protocols (PRISMA-P) 2015 statement.* Systematic Reviews, 2015. **4**: p. 1.
37. Higgins, J. and S. Green, *Cochrane Handbook for Systematic Reviews of Interventions*. 2008, Chichester, UK: John Wiley & Sons.
38. Sterne, J.A., et al., *ROBINS-I: a tool for assessing risk of bias in non-randomised studies of interventions.* British Medical Journal, 2016. **355**: p. i4919.
39. M., D., *Methods for the economic evaluation of health care programmes.* 2nd Edition ed, ed. O.U. Press. 1997.
40. Duval, S. and R. Tweedie, *Trim and fill: A simple funnel-plot-based method of testing and adjusting for publication bias in meta-analysis.* Biometrics, 2000. **56**(2): p. 455-63.
41. Guyatt, G.H., et al., *GRADE: an emerging consensus on rating quality of evidence and strength of recommendations.* British Medical Journal, 2008. **336**(7650): p. 924-6.

For peer review only

42. Silagy, C.A., P. Middleton, and S. Hopewell, *Publishing protocols of systematic reviews: comparing what was done to what was planned.* Journal of the American Medical Association, 2002. **287**(21): p. 2831-4.

For peer review only

445 **Figure Legends**

446 **Figure 1. Mapping the demonstrated benefits and risks of sharing EHR with patients across the six**
447 **domains of quality of care, as defined by the Institute of Medicine analytical framework.**
448 Subgroup analysis will be performed by domain of quality of care domain and by time scale.

For peer review only

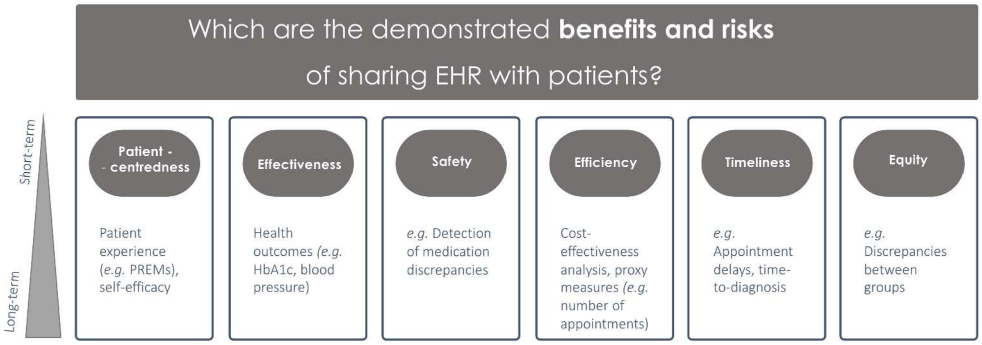

Figure 1. Mapping the demonstrated benefits and risks of sharing EHR with patients across the six domains of quality of care, as defined by the Institute of Medicine analytical framework. Subgroup analysis will be performed by domain of quality of care domain and by time scale.

326x170mm (300 x 300 DPI)

PRISMA-P (Preferred Reporting Items for Systematic review and Meta-Analysis Protocols) 2015 checklist: recommended items to address in a systematic review protocol*

Section and topic	Item No	Checklist item	Page
**ADMINISTRATIVE INFORMATION**			
Title:			
Identification	1a	Identify the report as a protocol of a systematic review	1, 2,
Update	1b	If the protocol is for an update of a previous systematic review, identify as such	Not applicable
Registration	2	If registered, provide the name of the registry (such as PROSPERO) and registration number	2
Authors:			
Contact	3a	Provide name, institutional affiliation, e-mail address of all protocol authors; provide physical mailing address of corresponding author	1
Contributions	3b	Describe contributions of protocol authors and identify the guarantor of the review	12
Amendments	4	If the protocol represents an amendment of a previously completed or published protocol, identify as such and list changes; otherwise, state plan for documenting important protocol amendments	Not applicable
Support:			
Sources	5a	Indicate sources of financial or other support for the review	12
Sponsor	5b	Provide name for the review funder and/or sponsor	12
Role of sponsor or funder	5c	Describe roles of funder(s), sponsor(s), and/or institution(s), if any, in developing the protocol	12
**INTRODUCTION**			
Rationale	6	Describe the rationale for the review in the context of what is already known	4-6
Objectives	7	Provide an explicit statement of the question(s) the review will address with reference to participants, interventions, comparators, and outcomes (PICO)	7
**METHODS**			
Eligibility criteria	8	Specify the study characteristics (such as PICO, study design, setting, time frame) and report characteristics (such as years considered, language, publication status) to be used as criteria for eligibility for the review	6,7,17
Information sources	9	Describe all intended information sources (such as electronic databases, contact with study authors, trial registers or other grey literature sources) with planned dates of coverage	6,7
Search strategy	10	Present draft of search strategy to be used for at least one electronic database, including planned limits, such that it could be repeated	6, 13-16

For peer review only

Study records:			
Data management	11a	Describe the mechanism(s) that will be used to manage records and data throughout the review	6-9
Selection process	11b	State the process that will be used for selecting studies (such as two independent reviewers) through each phase of the review (that is, screening, eligibility and inclusion in meta-analysis)	2,8
Data collection process	11c	Describe planned method of extracting data from reports (such as piloting forms, done independently, in duplicate), any processes for obtaining and confirming data from investigators	8
Data items	12	List and define all variables for which data will be sought (such as PICO items, funding sources), any pre-planned data assumptions and simplifications	7
Outcomes and prioritization	13	List and define all outcomes for which data will be sought, including prioritization of main and additional outcomes, with rationale	7
Risk of bias in individual studies	14	Describe anticipated methods for assessing risk of bias of individual studies, including whether this will be done at the outcome or study level, or both; state how this information will be used in data synthesis	8-9
Data synthesis	15a	Describe criteria under which study data will be quantitatively synthesised	8
	15b	If data are appropriate for quantitative synthesis, describe planned summary measures, methods of handling data and methods of combining data from studies, including any planned exploration of consistency (such as I^2, Kendall's τ)	8
	15c	Describe any proposed additional analyses (such as sensitivity or subgroup analyses, meta-regression)	8
	15d	If quantitative synthesis is not appropriate, describe the type of summary planned	8
Meta-bias(es)	16	Specify any planned assessment of meta-bias(es) (such as publication bias across studies, selective reporting within studies)	8
Confidence in cumulative evidence	17	Describe how the strength of the body of evidence will be assessed (such as GRADE)	8

*** It is strongly recommended that this checklist be read in conjunction with the PRISMA-P Explanation and Elaboration (cite when available) for important clarification on the items. Amendments to a review protocol should be tracked and dated. The copyright for PRISMA-P (including checklist) is held by the PRISMA-P Group and is distributed under a Creative Commons Attribution Licence 4.0.**

From: Shamseer L, Moher D, Clarke M, Ghersi D, Liberati A, Petticrew M, Shekelle P, Stewart L, PRISMA-P Group. Preferred reporting items for systematic review and meta-analysis protocols (PRISMA-P) 2015: elaboration and explanation. BMJ. 2015 Jan 2;349(jan02 1):g7647.

For peer review only

BMJ Open

Impact of sharing electronic health records with patients on the quality and safety of care: a systematic review and narrative synthesis protocol

Journal:	*BMJ Open*
Manuscript ID	bmjopen-2017-020387.R2
Article Type:	Protocol
Date Submitted by the Author:	21-May-2018
Complete List of Authors:	Neves, Ana Luisa; Imperial College London, Center for Health Policy, Institute of Global Health Innovation<br>Carter, Alexander; Imperial College London, Center for Health Policy, Institute of Global Health Innovation<br>Freise, Lisa; Imperial College London, Center for Health Policy, Institute of Global Health Innovation<br>Laranjo, Liliana; Macquarie University Faculty of Science, Australian Institute of Health Innovation; Universidade Nova de Lisboa, Centro de Investigação em Saúde Pública, Escola Nacional de Saúde Pública<br>Darzi, Ara; Imperial College London, Center for Health Policy, Institute of Global Health Innovation<br>Mayer, Erik; Imperial College London, Center for Health Policy, Institute of Global Health Innovation
**Primary Subject Heading**:	Health informatics
Secondary Subject Heading:	Evidence based practice, Health policy
Keywords:	Health informatics < BIOTECHNOLOGY & BIOINFORMATICS, Information technology < BIOTECHNOLOGY & BIOINFORMATICS, Quality in health care < HEALTH SERVICES ADMINISTRATION & MANAGEMENT, Health policy < HEALTH SERVICES ADMINISTRATION & MANAGEMENT, Health & safety < HEALTH SERVICES ADMINISTRATION & MANAGEMENT

SCHOLARONE™
Manuscripts

Impact of sharing electronic health records with patients on the quality and safety of care: a systematic review and narrative synthesis protocol

Authors: Ana Luisa Neves[1] (ALN) - ana.luisa.neves14@imperial.ac.uk, Alexander Carter (AC) - a.w.carter@imperial.ac.uk, Lisa Freise[1] (LF) - l.freise16@imperial.ac.uk, Liliana Laranjo[2] - liliana.laranjo@mq.edu.au, Ara Darzi[1] (AD) - a.darzi@imperial.ac.uk, Erik Mayer[1] (EM) - e.mayer@imperial.ac.uk

[1]Center for Health Policy, Institute of Global Health Innovation, Imperial College London, London, UK
[2]Australian Institute of Health Innovation, Centre for Health Informatics, Macquarie University, Sidney, Australia

For peer review only

Corresponding author

Name: Ana Luisa Neves

Address: Centre for Health Policy, Room 1035/7, QEQM Wing, St Mary's Campus, London W2 1NY

E-mail: ana.luisa.neves14@ic.ac.uk

Telephone: 02033127259

Fax number: 02033126309

Keywords [MeSH terms]: Electronic Health Records, Patient Access to Records, Patient-Centered Care, Quality of Health Care, Patient Safety

Word count: 2145 words

For peer review only - http://bmjopen.bmj.com/site/about/guidelines.xhtml

Abstract

Introduction: Providing patients with access to Electronic Health Records (EHR) has emerged as a promising solution to improve quality of care and safety. As the efforts to develop and implement EHR-based data sharing platforms mature and scale up worldwide, there is a need to evaluate the impact of these interventions and to weigh their relative risks and benefits, in order to inform evidence-based health policies. The aim of this work is to systematically characterise and appraise the demonstrated benefits and risks of sharing EHR with patients, by mapping them across the six domains of quality of care of the Institute of Medicine (IOM) analytical framework (*i.e.* patient-centredness, effectiveness, efficiency, timeliness, equity and safety).

Methods and analysis: CINAHL, Cochrane, Embase, HMIC, Medline/PubMed and PsycINFO databases will be searched from January 1997 to August 2017. Primary outcomes will include measures related with the six domains of quality of care of the IOM analytical framework. The quality of the studies will be assessed using the Cochrane Risk of Bias Tool, the ROBINS-I Tool and the Drummond's checklist. A narrative synthesis will be conducted for all included studies. Subgroup analysis will be performed by domain of quality of care domain and by time scale (*i.e.* short-, medium- or long-term impact). The body of evidence will be summarised in a Summary of Findings table and its strength assessed according to the GRADE criteria. This protocol was registered in PROSPERO (CRD42017070092).

Ethics and dissemination: This review does not require ethical approval as it will summarise published studies with non-identifiable data. This protocol complies with the Preferred Reporting Items for Systematic Review and Meta-Analysis Protocols (PRISMA-P) guidelines. Findings will be disseminated widely through peer-reviewed publication and conference presentations, and patient partners will be included in summarising the research findings into lay summaries and reports.

For peer review only

Strengths and limitations of this study

- Comprehensive characterisation of interventions sharing EHR with patients
- Summary and appraisal of existing evidence on the potential benefits and risks of these interventions, grouped by domain of quality of care
- Map the contribution of these interventions in short-, medium- and long-term time frames, in order to customise informed decisions in health policies
- Expected limitations include the heterogenous nature of the outcomes assessed and the potentially reduced sample size in subgroup analyses, which may negatively impact the statistical power in data synthesis.

For peer review only

Introduction

Although, in England, patients have had the legal right to access their health records since 1998, access to paper-based health records is mediated by health professionals and data controllers, through a cumbersome procedural process [1]. Additionally, as health information is fragmented between different organisations and levels of care, data access requests are often unable to provide a comprehensive health history record [2, 3].

In the last decade, electronic health records (EHR), have emerged as a promising solution to enhance patients' access to centralised medical information [4]. The adoption of EHR by primary care practices, hospitals and other healthcare organizations has steadily increased in the last years. In England, the percentage of General Practice surgeries that allowed patients to access their medical records online increased from 3% to 97% between April 2014 and February 2016 [5]. Patients' willingness and ability to access their health information through web portals is influenced by both individual (*i.e.* age, ethnicity, education level, health literacy and health status) and by health care delivery factors (*i.e.* provider endorsement and portal usability) [6, 7]. Various EHR-based platforms are currently used to share health information with patients, including direct online access, with or without patient-provider communication systems [8, 9] and health maintenance reminders [10, 11]. As these efforts mature and scale up worldwide, there is a need to evaluate the impact of interventions sharing EHR with patients, in order to weigh their relative risks and benefits and inform evidence-based health policies.

The Institute of Medicine (IOM) identified six domains of health care quality: patient-centredness, effectiveness, efficiency, timeliness, equity and safety [12]. Patient-centredness ensures that the care provided respects and responds to individual patient preferences, needs and values, thus incorporating these in clinical decisions [12, 13]. Health care shall provide evidence-based services, which can be ultimately expressed as improvements in health outcomes (*i.e.* effectiveness) [14], while ensuring patient safety (*i.e.* prevention of errors and adverse effects associated with health care) [12]. Other aspects of quality of delivery of care include the minimisation of the waste of human, physical or economical resources (*i.e.* efficiency), the reduction of waits and harmful delays (*i.e.* timeliness) and the reduction of avoidable differences on the delivery of care between different groups of health care users (*i.e.* equity) [12, 15, 16].

Providing patients with access to their health records has been linked to theorised benefits in four major domains of health care quality: patient-centredness, effectiveness, safety and efficiency [17-19]. However, despite the growing body of evidence on the theorised benefits of sharing EHR with patients on

For peer review only - http://bmjopen.bmj.com/site/about/guidelines.xhtml

these domains, there is still a considerable gap between the predicted and demonstrated benefits of these interventions [20].

In order to analyse the effect of providing patients access to their medical records on quality outcomes, Davis-Giardina T. *et al* performed a systematic review including studies published between 1970 and 2012 [21]. According to this work, a limited amount of evidence suggests that access to medical records improves patient satisfaction and enhances patient-provider communication [21]. Similarly, a systematic review from de Lusignan *et al.* reported that providing patients online access to their EHR increased convenience and satisfaction [22]. These findings are in line with the model proposed by Otte-Trojel T. *et al*, according to which sharing EHR with patients can improve both patient-provider communication and patient satisfaction, by increasing continuity of care and patient convenience, respectively [23].

Conversely, no clear benefits were found on effectiveness [21]. Until 2012, only a few studies evaluated the impact on effectiveness studies, most focusing on type 2 diabetes and with inconsistent results. Tenforde *et al* showed that providing access to medical records was associated with lower glycated haemoglobin A1c values [24]; however, no significant effect was found in three other studies assessing diabetes-specific effectiveness measures [25, 26]. One of the limitations of this review consists in the inclusion of studies evaluating the impact of sharing both electronic and paper-based health records - and this heterogeneity might mask potential specific benefits and risks of sharing EHR with patients. Furthermore, as pointed by the authors, the paucity of papers published up to that date resulted in a tendency to include small and methodologically less robust studies, thus increasing the risk of selective reporting and/or publication bias [21]. Mold F. *et al* also performed a systematic review assessing the impact of providing patients with access to their EHR; based in studies published between 1999 and 2012, this work found a positive influence in patient safety [27].

However, the authors were unable to find a consistent beneficial effect on efficiency measures (*i.e.* number of face-to-face visits and telephone appointments) in both reviews [21, 27]. While some studies reported an increase in the number of face-to-face consultations [8, 28] , others document a decrease [11, 29, 30]. Similarly, inconsistent results were found regarding the impact on telephone consultations: only one study reported a decline in the total number [31], whilst six other studies reported either no change or an increase [9, 28-30, 32, 33]. It is important to note, however, that most of the included studies assessing efficiency measures included in this review showed a high risk of bias, mostly related to either unclear or absent blinding methods [27].

The landmark reviews of Mold F. *et al* [27], Davis-Giardina T. *et al* [21], Ammenwerth E. *et al* [34] and Goldweig CL *et al* [7] provide a comprehensive characterisation of the literature published until 2013,

For peer review only

highlighting the paucity and the scientific limitations of the evidence published until that date. Although these reviews were unable to demonstrate clear benefits on efficiency and effectiveness measures, the debates around patients' rights and data ownership in the digital era, and the need to improve patient-centredness of health care delivery have acted as strong drivers to allocate resources to interventions and platforms aiming to share EHR with patients. As consequence of these efforts, it is plausible that studies performed in the last 5 years can provide further clarification for this evidence gap.

Furthermore, systematic reviews performed to the date do not address all domains of quality of care; in particular, the impact of sharing EHR with patients on timeliness or equity has not been addressed [7, 21, 27, 34, 35]. This is a particularly relevant gap in knowledge, given that interventions aimed at improving the quality of care do not necessarily improve all specific domains, and may even have a deleterious effect in some of them.

This review will expand on the above-mentioned work, in order to identify recent methodological and scientific progress until June 2017. Following the PRISMA-P checklist as guidance [36], we propose a systematic and reproducible strategy to query the literature on the demonstrated benefits and risks of sharing EHR with patients and map these results in a comprehensive framework of health care quality measures.

Research aims

The main objectives of this review are: 1) to systematically characterise interventions sharing EHR with patients; and 2) to assess the demonstrated risks and benefits of these interventions on patient-centredness, effectiveness, safety, efficiency, timeliness and equity, compared to usual care (no intervention). As secondary aim, we will map the contribution of these interventions in short-, medium- and long-term timeframes (Figure 1).

Methods and analysis

Search strategy

The search strategy will be performed using resources that enhance methodological transparency and improve the reproducibility of the results and evidence synthesis. A search of the literature from the last 20 years (January 1997 – August 2017) will be performed on CINAHL, Cochrane, Embase, HMIC, Medline/PubMed and PsycINFO. Search strings (Table 1) will combine free terms and controlled

For peer review only

vocabulary, whenever supported. We will also search grey literature sources, including registrations in the International Prospective Register of Systematic Reviews (PROSPERO), reports of relevant stakeholder organisations (NHS Digital, AMIA, eHealth at WHO, International Society for Telemedicine and eHealth), and conference proceedings (last 5 years) of several related conferences (American Medical Informatics Association [AMIA], MedInfo, Medicine 2.0, Medicine X), in order to identify possible additional studies that meet the inclusion criteria. Language restrictions will be applied and only articles in English will be included.

Study selection criteria

A summary of the participants, interventions, comparators and outcomes (PICO) considered, as well as the type of studies included, is provided in Table 2.

The systematic review will focus on studies on adult subjects, including both patients and carers (mean age of study sample ≥ 16 years). The systematic review does not focus on a particular disease area or health system setting as it intends to comprehensively characterise the scope of interventions sharing EHR with patients.

Studies assessing the impact of sharing EHR with patients, either isolated or as part of a multicomponent intervention, will be included. Included interventions will comprise: 1) web-based patient access to EHR; 2) EHR-based health reminders / messaging or 3) online patient-provider communication systems (health information exchange platforms). Studies focusing on health reminders only (not EHR-based) or appointment reminders will not be considered. The comparator will be 'no intervention' (*e.g.* usual care).

Primary outcomes will include any measure related to a) patient-centredness (*e.g.* patient-reported experience measures (PREMs), b) effectiveness (*e.g.* health outcomes); c) patient safety (*e.g.* identification of medication discrepancies); d) efficiency (*e.g.* economic evaluation measures and proxies; including service costs, number of consultations/admissions, e) timeliness (*e.g.* waiting lists, time-to-treatment) and f) equity (*e.g.* discrepancies in quality measures between different groups of patients) (Figure 1). Studies that only report cognitive outcomes (*e.g.* intent), motivational outcomes or other subjective psychological measures will be excluded. The types of study considered in this systematic review will be a) randomised controlled trials; b) cluster randomised trials; c) quasi-experimental studies; d) case-control studies, e) cohort studies and f) cost-effectiveness studies. The reference lists of systematic reviews identified in this search will also be screened to ensure all eligible studies are captured.

For peer review only

Screening and data extraction

Quantitative studies will be independently assessed by three reviewers and reported using the Preferred Reporting Items for Systematic Reviews and Meta-Analyses (PRISMA-P) flow diagram [36]. Initial screening of studies will be based on the information contained in their titles and abstracts and will be conducted by two independent investigators. Full-paper screening will be conducted by the same independent investigators. Cohen's kappa will be used to measure inter-coder agreement in each screening phase. When there are doubts regarding inclusion or exclusion, a third investigator will be involved in the decision. Two independent investigators will extract information from the included studies into a standardized form. The data collected for each study will include: name of the first author, year of publication, technology, intervention components and characteristics, study duration, participants' and setting characteristics, outcomes and retention rates. Two investigators will review the abstraction form for consistency. Disagreements will be resolved by a third investigator.

Quality assessment

The quality of randomised controlled trials and cluster randomized trials will be assessed using the Cochrane Risk of Bias Tool [37], that assesses the following study-level aspects: a) randomisation sequence allocation; b) allocation concealment; c) blinding; d) completeness of outcome data and e) selective outcome reporting. The quality of non-randomised intervention studies (*i.e.* case control, cohort, quasi-experimental) will be appraised using the ROBINS-I tool, which assesses bias due to a) confounding, b) selection of participants, c) classification of interventions, d) deviations from intended interventions, e) missing data, f) measurement of outcomes and g) selection of reported results [38]. For cost-effectiveness studies, the Drummond's checklist for assessing economic evaluations will be used [39]. Two independent reviewers will score the selected studies and disagreements will be resolved by a third person.

The risk of bias for each outcome across individual studies will be summarised as a narrative statement, and supported by a risk of bias table. A review-level narrative summary of the risk of bias will also be provided.

For peer review only

Descriptive analysis and meta-analysis

Planned subgroup analysis will be performed by domain of quality of care (IOM framework) and by time scale (*i.e.* short-, medium- or long-term impact). For studies with a high or unclear risk of bias, defined as high or unclear risk in 50% or more of the quality assessment outcomes, a narrative description of the risk of bias will be provided. Risk of bias assessments will be incorporated into synthesis by performing sensitivity analysis (*i.e.* limiting to studies at lowest risk of bias in a secondary analysis). Depending on the amount of information retrieved, subgroup analysis will also be performed for specific diseases.

A narrative synthesis will be conducted for all the included studies. Parallel-group trials that are deemed comparable in relevant ways will be pooled together for a summary effect. Whenever possible, continuous and dichotomous outcomes will be pooled together for meta-analysis purposes. All effect sizes will be transformed into a common metric, in order to make them comparable across studies — the bias-corrected standardized difference in means (Hedges' g) — classified as positive when in favor of the intervention and negative when in favor of the control. Heterogeneity will be assessed using I^2. The presence of publication bias will be evaluated by use of a funnel plot and the Duval and Tweedie's trim and fill method [40].

The body of evidence will be summarised in a Summary of Findings table and the strength of the body of evidence will be assessed according to GRADE criteria [41].

Patient and public involvement

Our research question emerged from the implementation evaluation of the Care Information Exchange (CIE), a pilot web portal/patient-controlled EHR happening across a 2.4 million population in North West London. CIE implementation evaluation was shaped by its steering group, which included lay partners, and their perspectives reinforced that our research question was relevant and aligned with patients' interest.

Patients were not directly involved in the design of this study. As this is a protocol for a systematic review and no participant recruitment will take place, their involvement on the recruitment and dissemination of findings to participants was not applicable.

However, patient partners will be included in the interpretation of our results, in the co-development of a dissemination strategy, and in summarising the research findings into lay summaries and reports, in order to raise awareness and stimulate public participation on this topic.

For peer review only

Amendments

Any amendments to this protocol will be documented with reference to saved searches and analysis methods, which will be recorded in bibliographic databases (Ovid), Endnote, and Excel templates for data collection and synthesis.

For peer review only

Discussion

As the implementation of interventions to share EHR with patients scales up worldwide, the systematic evaluation of their impact emerges as a priority research topic.

One of the strengths of the proposed study is to apply a reproducible and transparent procedure for systematic review of the literature. In this protocol, we clearly describe the types of studies, participants, interventions, and outcomes that will be included, as well as the data sources, search strategy, data extraction methods (including quality assessment) and methods of combining data [42]. By publishing the research protocol, we reinforce the clarity of the strategy and minimise the risk of bias, namely selective outcome reporting [37]. Second, we will focus solely on the impact of EHR-based studies, increasing the sensitivity to detect specific benefits of this type of intervention. Third, for the first time, we aim to comprehensively evaluate both the benefits and risks of these interventions in a wide range of domains of quality of care, as defined by the IOM, and in diverse time frames. This results shall provide high-level information to inform, support and customise decisions in health policies.

Potential limitations of this study include the heterogeneity of measures and outcomes evaluated and the potentially reduced number of studies in subgroup analyses, which may negatively influence the statistical power in data synthesis.

For peer review only

Acknowledgements

We thank Jacqueline Cousins (Library Manager and Liaison Librarian at Imperial College London) for the support improving the composition of the search terms and procedural aspects of the search strategy. We would also like to acknowledge Dr Søren Rud Kristensen (Senior Lecturer in Health Economics at the Centre for Health Policy at the Institute of Global Health Innovation) and Dr Joachim Marti (Senior Lecturer in Health Economics at the Centre for Health Policy at the Institute of Global Health Innovation) for their advices regarding the quality appraisal of cost-effectiveness studies, and Miss Anna Lawrence-Jones (Patient and Public Involvement and Engagement Manager at Imperial National Institute for Health Research (NIHR) Patient Safety Translation Research Centre) for the useful discussions around public and patient involvement.

Ethics and dissemination

This review does not require ethical approval as it will summarise published studies with non-identifiable data. This protocol complies with the PRISMA-P guidelines. Findings will be disseminated widely through peer-reviewed publication and conference presentations. Patient partners will be included in the co-development of our dissemination strategy, and in summarising the research findings into lay summaries and reports.

Authors' contributions

ALN, AC and EKM conceptualised this research. ALN, AC and LL designed the protocol. ALN and LF defined the concepts and search items. EKM and AD contributed to the conceptualisation and commented on the multiple versions of the protocol. The manuscript was written by ALN with contributions from all authors.

Funding statement

This work is supported by the Sowerby Program and by the National Institute for Health Research (NIHR) Patient Safety Translation Research Centre. The views of the authors do not necessarily reflect those of the NHS, NIHR or the Department of Health.

Competing interests' statement

For peer review only

We declare no conflict(s) of interest associated with this research.

Data sharing statement

No additional data relevant to this protocol are available for distribution. All relevant data and search terms are published in this manuscript.

For peer review only

Table 1. Concepts and search items

**Database**	**Search items**
**CINAHL *via* EBSCO**	1. (((electronic* or online or on-line or digital*) N1 (health record* or medical record* or personal record* or patient record*)) or EHR# or EMR# or ephr#)<br>2. ((information or data) N4 (shar* or exchang*)) or HIE or HIEs or access*)<br>3. #1 and #2<br>4. (((experience or satisfaction) N4 (patient* or consumer* or client* or survey or questionnaire*)) or PREM* or patient-reported experience measure*)<br>5.(effectiveness or health outcome*)<br>6. (patient N1 (safety or harm)) or safety manag* or accident prevent* or error* or medication reconcil* or near miss*<br>7. (efficiency or economic* or cost* or expenditure* or charge* or fee*1 or (number N1 appointment*) or (number adj2 admission*) or (number N1 telephone visit*))<br>8. waiting lists or timeliness or time-to-treatment<br>9. health equity<br>10. #4 or #5 or #6 or #7 or #8 or #9<br>13. 3 and 10
**Cochrane *via* url:** http://onlinelibrary.wiley.com/cochranelibrary/search/advanced	1. (((electronic* or online or on-line or digital*) near/1 (health record* or medical record* or personal record* or patient record*)) or EHR or EHRs or EMR or EMRs or ephr or ephrs)<br>2. Electronic Health Records [MesH]<br>3. #1 or #2<br>4. (((information or data) near/4 (shar* or exchang*)) or HIE or HIES or access*)<br>5. Information Dissemination [MesH]<br>6. #4 or #5<br>7. #3 and #6<br>8. (((experience or satisfaction) near/4 (patient* or consumer* or client* or survey or questionnaire*)) or PREM* or patient-reported experience measure*)<br>9. (effectiveness or health outcome*)<br>10. (patient near/1 safety) or (patient near/1 harm) or safety manag* or accident prevent* or error* or medication reconcil*<br>11. (efficiency or economic* or cost* or expenditure* or charge* or fee* or (number near/1 appointment*) or (number near/1 admission*) or (number near/1 telephone visit*))<br>12. time-to-treatment or timeliness<br>13. waiting lists [MesH]<br>14. health equity [MesH]<br>15. #8 or #9 or #10 or #11 or #12 or #13 or #14

For peer review only

	17. 7 and 15
**Embase *via* Ovid**	1. (((electronic* or online or on-line or digital*) adj2 (health record* or medical record* or personal record* or patient record*)) or EHR? or EMR? or ephr?)<br>2. Electronic health record/<br>3. 1 or 2<br>4. (((information or data) adj5 (shar* or exchang*)) or HIE*2 or access*)<br>5. Information dissemination/<br>6. 4 or 5<br>7. 3 and 6<br>8. (((experience or satisfaction) adj5 (patient* or consumer* or client* or survey or questionnaire*)) or PREM* or patient-reported experience measure*)<br>9. (effectiveness or health outcome*) 10. (patient adj2 (safety or harm)) or safety manag* or accident prevent* or error* or medication reconcil* or near miss*<br>11. (efficiency or economic* or cost* or expenditure* or charge* or fee*1 or (number adj2 appointment*) or (number adj2 admission*) or (number adj2 telephone visit*))<br>12. waiting list* or time to treatment/ or timeliness<br>13. health equity/<br>14. 8 or 9 or 10 11 or 12 or 13<br>15. 7 and 14
**HMIC *via* Ovid**	1. (((electronic* or online or on-line or digital*) adj2 (health record* or medical record* or personal record* or patient record*)) or EHR? or EMR? or ephr?)<br>2. Electronic patient records/<br>3. 1 or 2<br>4. (((information or data) adj5 (shar* or exchang*)) or HIE*2 or access*)<br>5. Information exchange/<br>6. 4 or 5<br>7. 3 and 6<br>8. (((experience or satisfaction) adj5 (patient* or consumer* or client* or survey or questionnaire*)) or PREM* or patient-reported experience measure*)<br>9. (effectiveness or health outcome*) 10. (patient adj2 (safety or harm)) or safety manag* or accident prevent* or error* or medication reconcil* or near miss*<br>11. (efficiency or economic* or cost* or expenditure* or charge* or fee*1 or (number adj2 appointment*) or (number adj2 admission*) or (number adj2 telephone visit*))<br>12. waiting lists/ or patient waiting time or timeliness<br>13. health inequalities/ or equity

For peer review only

	14. 8 or 9 or 10 or 11 or 12 or 13<br>15. 7 and 14
**Medline via Ovid**	1. (((electronic* or online or on-line or digital*) adj2 (health record* or medical record* or personal record* or patient record*)) or EHR? or EMR? or ephr?)<br>2. Electronic Health Records/<br>3. 1 or 2<br>4. (((information or data) adj5 (shar* or exchang*)) or HIE*2 or access*<br>5. Information Dissemination/<br>6. 4 or 5<br>7. 3 and 6<br>8. (((experience or satisfaction) adj5 (patient* or consumer* or client* or survey or questionnaire*)) or PREM* or patient-reported experience measure*)<br>9. (effectiveness or health outcome*)<br>10. ((patient adj2 (safety or harm)) or safety manag* or accident prevent* or error* or medication reconcil* or near miss*)<br>11. (efficiency or economic* or cost* or expenditure* or charge* or fee*1 or (number adj2 appointment*) or (number adj2 admission*) or (number adj2 telephone visit*))<br>12. Waiting Lists/ or Time-to-treatment/ or timeliness<br>13. Health Equity/<br>14. 8 or 9 or 10 or 11 or 12 or 13 15. 7 and 14
**PsycINFO *via* Ovid**	1. (((electronic* or online or on-line or digital*) adj2 (health record* or medical record* or personal record* or patient record*)) or EHR? or EMR? or ephr?)<br>2. (((information or data) adj5 (shar* or exchang*)) or HIE*2 or access*)<br>3. 1 and 2<br>4. (((experience or satisfaction) adj5 (patient* or consumer* or client* or survey or questionnaire*)) or PREM* or patient-reported experience measure*)<br>5. (effectiveness or health outcome*)<br>6. (patient adj2 (safety or harm)) or safety manag* or accident prevent* or error* or medication reconcil* or near miss*<br>7. (efficiency or economic* or cost* or expenditure* or charge* or fee*1 or (number adj2 appointment*) or (number adj2 admission*) or (number adj2 telephone visit*))<br>8. waiting list* or time-to-treatment or timeliness<br>9. equity or health disparities/<br>10. 4 or 5 or 6 or 7 or 8 or 9<br>11. 3 and 10

Search themes (facets) and terms derived for each theme relating to the use of EHR and predicted benefits (patient experience, effectiveness and efficiency)

For peer review only

Table 2. Inclusion and exclusion criteria

	Inclusion criteria	Exclusion criteria
**Population**	Adult subjects (patients and carers)	Individuals 16 years of age and under (e.g. mean age of study sample <16)
**Intervention**	Electronic health record-based interventions, including:<br><br>• Patient access to EHR<br>• EHR-based reminders / messaging<br>• Unidirectional or bidirectional online patient-provider communication systems (care information exchange platforms);	• Health reminders only
**Comparison**	No intervention (*e.g.* usual care)	
**Outcome**	Any measure related to a) patient-centredness (*e.g.* PREMs), b) effectiveness (*e.g.* health outcomes)*;* c) patient safety (*e.g.* identification of medication discrepancies); d) efficiency (*e.g.* economic evaluation measures and proxies; including service costs, number of consultations/admissions, e) timeliness (*e.g.* waiting lists, time-to-treatment) or f) equity (*e.g.* discrepancies in quality measures between different groups of patients).	Studies that only report cognitive outcomes (*e.g.* intention to), motivational outcomes or other subjective psychological measures
**Study type**	Randomised controlled trials, cluster randomised trials, quasi-experimental, case-control, cohort studies, cost-effectiveness	

For peer review only

Reference

1. Dimond, B., *Rights to information access under the Data Protection Act.* British Journal of Nursing, 2005. **14**(14): p. 774-6.
2. Stange, K.C., *The problem of fragmentation and the need for integrative solutions.* Annals of Family Medicine, 2009. **7**(2): p. 100-3.
3. Roukema, J., et al., *Paper versus computer: feasibility of an electronic medical record in general pediatrics.* Pediatrics, 2006. **117**(1): p. 15-21.
4. Vest, J.R., *Health information exchange: national and international approaches.* Advances in Health Care Management, 2012. **12**: p. 3-24.
5. Houses of Parliament, *EHR House of Parliament Report.* 2016.
6. Irizarry, T., A. DeVito Dabbs, and C.R. Curran, *Patient Portals and Patient Engagement: A State of the Science Review.* Journal of Medical Internet Research, 2015. **17**(6): p. e148.
7. Goldzweig, C.L., et al., *Electronic patient portals: evidence on health outcomes, satisfaction, efficiency, and attitudes: a systematic review.* Annals of Internal Medicine, 2013. **159**(10): p. 677-87.
8. Delbanco, T., et al., *Inviting patients to read their doctors' notes: a quasi-experimental study and a look ahead.* Annals of Internal Medicine, 2012. **157**(7): p. 461-70.
9. Katz, S.J., N. Nissan, and C.A. Moyer, *Crossing the digital divide: evaluating online communication between patients and their providers.* The American Journal of Managed Care, 2004. **10**(9): p. 593-8.
10. Wright, A., et al., *Randomized controlled trial of health maintenance reminders provided directly to patients through an electronic PHR.* Journal of General Internal Medicine, 2012. **27**(1): p. 85-92.
11. Nagykaldi, Z., et al., *Impact of a Wellness Portal on the delivery of patient-centered preventive care.* Journal of the American Board of Family Medicine, 2012. **25**(2): p. 158-67.
12. America, I.o.M.C.o.Q.o.H.C.i., *Crossing the Quality Chasm: A New Health System for the 21st Century*. 2001, Washington (DC): National Academies Press.
13. Scholl, I., et al., *An integrative model of patient-centeredness - a systematic review and concept analysis.* PloS One, 2014. **9**(9): p. e107828.
14. Arah, O.A., et al., *Conceptual frameworks for health systems performance: a quest for effectiveness, quality, and improvement.* Journal of the International Society for Quality in Health Care, 2003. **15**(5): p. 377-98.
15. Hussey, P.S., et al., *A systematic review of health care efficiency measures.* Health services Research, 2009. **44**(3): p. 784-805.
16. World Health Organisation, *Health Systems: Equity, 2017.* Available at http://www.who.int/healthsystems/topics/equity/en/. Last access: 26th March 2018.
17. Wagner, K.A., F.W. Lee, and J.P. Glaser, *Healthcare information systems: A practical approach for health care management.*, J.W. Sons. 2013.
18. Department of Health, *The power of information: giving people control of the health and care information they need,* 2012. Available at: https://www.gov.uk/government/publications/giving-people-control-of-the-health-and-care-information-they-need. Last access: 26th March 2018.
19. Ross, S.E. and C.-T. Lin, *The effects of promoting patient access to medical records: a review.* Journal of the American Medical Informatics Association, 2003. **10**(2): p. 129-38.
20. Jilka, S.R., et al., *"Nothing About Me Without Me": An Interpretative Review of Patient Accessible Electronic Health Records.* Journal of Medical Internet Research, 2015. **17**(6): p. e161.

For peer review only

21. Davis Giardina, T., et al., *Patient access to medical records and healthcare outcomes: a systematic review.* Journal of the American Medical Informatics Association, 2014. **21**(4): p. 737-41.
22. de Lusignan, S., et al., *Patients' online access to their electronic health records and linked online services: a systematic interpretative review.* BMJ Open, 2014. **4**(9): p. e006021.
23. Otte-Trojel, T., et al., *How outcomes are achieved through patient portals: a realist review.* Journal of the American Medical Informatics Association, 2014. **21**(4): p. 751-7.
24. Tenforde, M., et al., *The association between personal health record use and diabetes quality measures.* Journal of General Internal Medicine, 2012. **27**(4): p. 420-4.
25. McCarrier, K.P., et al., *Web-based collaborative care for type 1 diabetes: a pilot randomized trial.* Diabetes Technology & Therapeutics, 2009. **11**(4): p. 211-7.
26. Grant, R.W., et al., *Practice-linked online personal health records for type 2 diabetes mellitus: a randomized controlled trial.* Archives of Internal Medicine, 2008. **168**(16): p. 1776-82.
27. Mold, F., et al., *Patients' online access to their electronic health records and linked online services: a systematic review in primary care.* The British Journal of General Practice, 2015. **65**(632): p. e141-51.
28. Palen, T.E., et al., *Association of online patient access to clinicians and medical records with use of clinical services.* Journal of the American Medical Association, 2012. **308**(19): p. 2012-9.
29. Bergmo, T.S., et al., *Electronic patient-provider communication: will it offset office visits and telephone consultations in primary care?* International Journal of Medical Informatics, 2005. **74**(9): p. 705-10.
30. Zhou, Y.Y., et al., *Patient access to an electronic health record with secure messaging: impact on primary care utilization.* The American Journal of Managed Care, 2007. **13**(7): p. 418-24.
31. Kummervold, P.E., et al., *[Patient-physician interaction over the internet].* Tidsskr Nor Laegeforen. 2004 Oct 21;124(20):2633-6.
32. Lin, C.-T., et al., *An Internet-based patient-provider communication system: randomized controlled trial.* Journal of Medical Internet Research, 2005. **7**(4): p. e47.
33. Katz, S.J., et al., *Effect of a triage-based E-mail system on clinic resource use and patient and physician satisfaction in primary care: a randomized controlled trial.* Journal of General Internal Medicine, 2003. **18**(9): p. 736-44.
34. Ammenwerth, E., P. Schnell-Inderst, and A. Hoerbst, *The impact of electronic patient portals on patient care: a systematic review of controlled trials.* Journal of Medical Internet research, 2012. **14**(6): p. e162.
35. Ammenwerth, E., et al., *Adult patient access to electronic health records (Protocol).* Cochrane Database of Systematic Reviews 2017(6): p. CD012707.
36. Moher, D., et al., *Preferred reporting items for systematic review and meta-analysis protocols (PRISMA-P) 2015 statement.* Systematic Reviews, 2015. **4**: p. 1.
37. Higgins, J. and S. Green, *Cochrane Handbook for Systematic Reviews of Interventions*. 2008, Chichester, UK: John Wiley & Sons.
38. Sterne, J.A., et al., *ROBINS-I: a tool for assessing risk of bias in non-randomised studies of interventions.* British Medical Journal, 2016. **355**: p. i4919.
39. M., D., *Methods for the economic evaluation of health care programmes.* 2nd Edition ed, ed. O.U. Press. 1997.
40. Duval, S. and R. Tweedie, *Trim and fill: A simple funnel-plot-based method of testing and adjusting for publication bias in meta-analysis.* Biometrics, 2000. **56**(2): p. 455-63.
41. Guyatt, G.H., et al., *GRADE: an emerging consensus on rating quality of evidence and strength of recommendations.* British Medical Journal, 2008. **336**(7650): p. 924-6.

For peer review only

42. Silagy, C.A., P. Middleton, and S. Hopewell, *Publishing protocols of systematic reviews: comparing what was done to what was planned.* Journal of the American Medical Association, 2002. **287**(21): p. 2831-4.

For peer review only

449 **Figure Legends**

450 **Figure 1. Mapping the demonstrated benefits and risks of sharing EHR with patients across the six**
451 **domains of quality of care, as previously defined by the Institute of Medicine analytical framework**
452 **[12].** Subgroup analysis will be performed by domain of quality of care domain and by time scale.

For peer review only

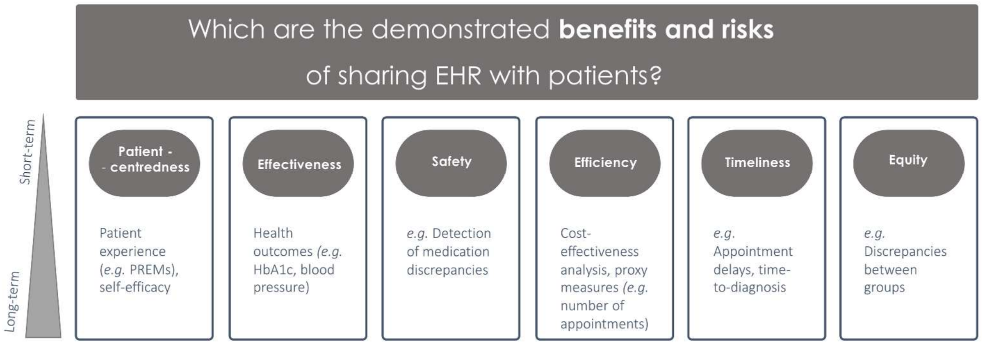

Figure 1. Mapping the demonstrated benefits and risks of sharing EHR with patients across the six domains of quality of care, as defined by the Institute of Medicine analytical framework. Subgroup analysis will be performed by domain of quality of care domain and by time scale.

326x170mm (300 x 300 DPI)

review only

PRISMA-P (Preferred Reporting Items for Systematic review and Meta-Analysis Protocols) 2015 checklist: recommended items to address in a systematic review protocol*

Section and topic	Item No	Checklist item	Page
**ADMINISTRATIVE INFORMATION**			
Title:			
Identification	1a	Identify the report as a protocol of a systematic review	1, 2,
Update	1b	If the protocol is for an update of a previous systematic review, identify as such	Not applicable
Registration	2	If registered, provide the name of the registry (such as PROSPERO) and registration number	2
Authors:			
Contact	3a	Provide name, institutional affiliation, e-mail address of all protocol authors; provide physical mailing address of corresponding author	1
Contributions	3b	Describe contributions of protocol authors and identify the guarantor of the review	12
Amendments	4	If the protocol represents an amendment of a previously completed or published protocol, identify as such and list changes; otherwise, state plan for documenting important protocol amendments	Not applicable
Support:			
Sources	5a	Indicate sources of financial or other support for the review	12
Sponsor	5b	Provide name for the review funder and/or sponsor	12
Role of sponsor or funder	5c	Describe roles of funder(s), sponsor(s), and/or institution(s), if any, in developing the protocol	12
**INTRODUCTION**			
Rationale	6	Describe the rationale for the review in the context of what is already known	4-6
Objectives	7	Provide an explicit statement of the question(s) the review will address with reference to participants, interventions, comparators, and outcomes (PICO)	7
**METHODS**			
Eligibility criteria	8	Specify the study characteristics (such as PICO, study design, setting, time frame) and report characteristics (such as years considered, language, publication status) to be used as criteria for eligibility for the review	6,7,17
Information sources	9	Describe all intended information sources (such as electronic databases, contact with study authors, trial registers or other grey literature sources) with planned dates of coverage	6,7
Search strategy	10	Present draft of search strategy to be used for at least one electronic database, including planned limits, such that it could be repeated	6, 13-16

For peer review only

Study records:			
Data management	11a	Describe the mechanism(s) that will be used to manage records and data throughout the review	6-9
Selection process	11b	State the process that will be used for selecting studies (such as two independent reviewers) through each phase of the review (that is, screening, eligibility and inclusion in meta-analysis)	2,8
Data collection process	11c	Describe planned method of extracting data from reports (such as piloting forms, done independently, in duplicate), any processes for obtaining and confirming data from investigators	8
Data items	12	List and define all variables for which data will be sought (such as PICO items, funding sources), any pre-planned data assumptions and simplifications	7
Outcomes and prioritization	13	List and define all outcomes for which data will be sought, including prioritization of main and additional outcomes, with rationale	7
Risk of bias in individual studies	14	Describe anticipated methods for assessing risk of bias of individual studies, including whether this will be done at the outcome or study level, or both; state how this information will be used in data synthesis	8-9
Data synthesis	15a	Describe criteria under which study data will be quantitatively synthesised	8
	15b	If data are appropriate for quantitative synthesis, describe planned summary measures, methods of handling data and methods of combining data from studies, including any planned exploration of consistency (such as I^2, Kendall's τ)	8
	15c	Describe any proposed additional analyses (such as sensitivity or subgroup analyses, meta-regression)	8
	15d	If quantitative synthesis is not appropriate, describe the type of summary planned	8
Meta-bias(es)	16	Specify any planned assessment of meta-bias(es) (such as publication bias across studies, selective reporting within studies)	8
Confidence in cumulative evidence	17	Describe how the strength of the body of evidence will be assessed (such as GRADE)	8

*** It is strongly recommended that this checklist be read in conjunction with the PRISMA-P Explanation and Elaboration (cite when available) for important clarification on the items. Amendments to a review protocol should be tracked and dated. The copyright for PRISMA-P (including checklist) is held by the PRISMA-P Group and is distributed under a Creative Commons Attribution Licence 4.0.**

From: Shamseer L, Moher D, Clarke M, Ghersi D, Liberati A, Petticrew M, Shekelle P, Stewart L, PRISMA-P Group. Preferred reporting items for systematic review and meta-analysis protocols (PRISMA-P) 2015: elaboration and explanation. BMJ. 2015 Jan 2;349(jan02 1):g7647.

For peer review only
